# Oncogenic cooperation between *TCF7-SPI1* and *NRAS*(G12D) requires β-catenin activity to drive T-cell acute lymphoblastic leukemia

**DOI:** 10.1038/s41467-021-24442-9

**Published:** 2021-07-06

**Authors:** Quentin Van Thillo, Jolien De Bie, Janith A. Seneviratne, Sofie Demeyer, Sofia Omari, Anushree Balachandran, Vicki Zhai, Wai L. Tam, Bram Sweron, Ellen Geerdens, Olga Gielen, Sarah Provost, Heidi Segers, Nancy Boeckx, Glenn M. Marshall, Belamy B. Cheung, Kiyotaka Isobe, Itaru Kato, Junko Takita, Timothy G. Amos, Ira W. Deveson, Hannah McCalmont, Richard B. Lock, Ethan P. Oxley, Maximilian M. Garwood, Ross A. Dickins, Anne Uyttebroeck, Daniel R. Carter, Jan Cools, Charles E. de Bock

**Affiliations:** 1 Department of Human Genetics, KU Leuven, Leuven, Belgium; 2 Center for Cancer Biology, VIB, Leuven, Belgium; 3 Leuvens Kanker Instituut (LKI), KU Leuven – UZ Leuven, Leuven, Belgium; 4 Center for Human Genetics, UZ Leuven, Leuven, Belgium; 5 Children’s Cancer Institute, UNSW Sydney, Lowy Cancer Research Centre, Sydney, NSW Australia; 6 School of Women’s and Children’s Health, UNSW Sydney, Sydney, NSW Australia; 7 Technology Innovation Lab, VIB, Gent, Belgium; 8 Department of Oncology, KU Leuven, Leuven, Belgium; 9 Department of Pediatric Hemato-Oncology, UZ Leuven, Leuven, Belgium; 10 Department of Laboratory Medicine, UZ Leuven, Leuven, Belgium; 11 Kids Cancer Centre, Sydney Children’s Hospital, Randwick, NSW Australia; 12 Department of Pediatrics, Graduate School of Medicine, Kyoto University, Kyoto, Japan; 13 Kinghorn Centre for Clinical Genomics, Garvan Institute of Medical Research, Sydney, NSW Australia; 14 St Vincent’s Clinical School, Faculty of Medicine, UNSW Sydney, Sydney, NSW Australia; 15 Australian Centre for Blood Diseases, Monash University, Melbourne, VIC Australia; 16 School of Biomedical Engineering, University of Technology, Sydney, NSW Australia

**Keywords:** Cancer genetics, Acute lymphocytic leukaemia

## Abstract

*Spi-1 Proto-Oncogene (SPI1)* fusion genes are recurrently found in T-cell acute lymphoblastic leukemia (T-ALL) cases but are insufficient to drive leukemogenesis. Here we show that *SPI1* fusions in combination with activating *NRAS* mutations drive an immature T-ALL in vivo using a conditional bone marrow transplant mouse model. Addition of the oncogenic fusion to the *NRAS* mutation also results in a higher leukemic stem cell frequency. Mechanistically, genetic deletion of the β-catenin binding domain within *Transcription factor 7* (*TCF7)-SPI1* or use of a TCF/β-catenin interaction antagonist abolishes the oncogenic activity of the fusion. Targeting the *TCF7-SPI1* fusion in vivo with a doxycycline-inducible knockdown results in increased differentiation. Moreover, both pharmacological and genetic inhibition lead to down-regulation of *SPI1* targets. Together, our results reveal an example where *TCF7-SPI1* leukemia is vulnerable to pharmacological targeting of the TCF/β-catenin interaction.

## Abstract (web summary)

SPI1 fusion genes in T-cell acute lymphoblastic leukemia (T-ALL) are commonly found with co-occurring NRAS mutations. Here, the authors show that the combination of these oncogenes is necessary to drive T-ALL in a murine model and that the oncogenic activity of the SPI1 fusion is dependent on β-catenin.

## Introduction

T-cell acute lymphoblastic leukemia or T-ALL is a hematopoietic malignancy of T-cell precursor cells that primarily affects young children. Chromosomal rearrangements are present in the majority of T-ALL patients, resulting in the ectopic expression of transcription factors, including T-cell acute lymphocytic leukemia protein 1 (TAL1), T-cell leukemia homeobox protein 1 and 3 (TLX1 and TLX3) and NK2 homeobox 1 (NKX2-1). Other typical pathways involved in the pathogenesis of T-ALL include the NOTCH1, Janus kinase (JAK)/signal transducer of activation and transcription (STAT) and RAS signaling pathways^1–5^. Often multiple cooperating hits are needed to induce leukemia^6–8^.

Seki et al. recently reported that up to 4% of pediatric T-ALL patients carry gene fusions involving the *SPI1* locus on chromosome 11 (also known as PU.1) with different fusion partners, and that patients harboring such *SPI1* fusions represent a distinct subgroup of T-ALL cases with significantly shorter overall survival^9^. While *SPI1* (PU.1) is an important transcription factor in all hematopoietic cells, *TCF7* (located on the short arm of chromosome 5) encodes TCF1, a T-cell specific transcriptional activator that is highly expressed in the earliest thymic progenitors and controls chromatin accessibility of T-cell regulatory elements^10,11^. Dysregulation of PU.1 contributes to several types of leukemia^12–18^ and forced overexpression of *SPI1* in T-cell progenitors is known to inhibit T-cell differentiation^19–21^. Conversely, *TCF7* knock-out also leads to a T-cell differentiation block^22^.

In normal T-cell development, NOTCH-signaling plays an important role in downregulating PU.1 and upregulating TCF1 expression during progression from the double negative (DN) 2 to DN3 T-cell stage^20,23,24^. To evaluate the role of *SPI1* fusion genes in T-ALL, Seki et al. expressed distinct fusion transcripts in mouse stem/progenitor cells and documented an increased T-cell proliferation and a maturation block at the DN3 stage upon *SPI1* or *SPI1* fusion expression. Constitutive expression of *TCF7-SPI1* alone, however, was not found to cause leukemia development in a mouse bone marrow transplant model^9^. Significantly, whilst *TCF7-SPI1* fusions are insufficient for frank T-cell leukemia development, these clinical cases are often coincident with NRAS mutations suggesting the potential for oncogenic cooperation. Here we show that *TCF7-SPI1* fusions and the *NRAS*(G12D) activating mutation cooperate to form an immature T-ALL in vivo that is absent when each is expressed alone. Moreover, using a genetic approach we show this cooperation absolutely requires the presence of the β-catenin interaction domain in the *TCF7-SPI1* fusion with genetic loss of the fusion resulting in immunophenotypic differentiation. We also provide proof-of-principle that this is phenocopied through small molecule antagonism of the interaction between β-catenin and TCF1 of the *TCF7-SPI1* fusion.

## Results

### Identification of a *TCF7-SPI1* positive and *NRAS* mutant T-ALL case

**Fig. 1:**
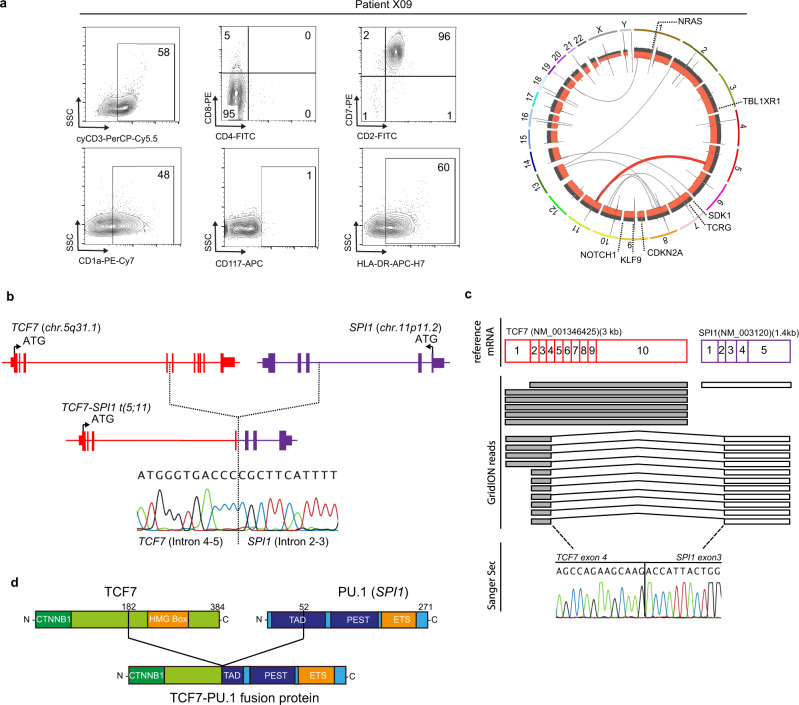
Identification and characterization of the *TCF7-SPI1* fusion in patient X09 with T-cell acute lymphoblastic leukemia. **a** Left panel: flow cytometry staining of the X09 patient sample for cyCD3, CD1a, CD4, CD8, CD117, CD2, CD7, HLA-DR. Right panel: Circos plot showing the translocation between chromosomes 5 and 11 in the X09 patient. Additional mutations are shown with their respective chromosomal location. **b** Scheme of the t(5;11) translocation between *TCF7* and *SPI1*. The breakpoints are located at *TCF7* intron 4-5 (Chr. 5) and *SPI1* intron 2–3 (Chr. 11). **c** Long read nanopore sequencing confirming the translocation with the resulting GridION reads shown schematically alongside Sanger sequencing confirmation of the breakpoint region. **d** Schematic representation of the resulting TCF1-PU.1 (*TCF7-SPI1*) fusion protein. The different domains are shown. CTNNB1: β-catenin binding domain; TAD: trans-activating domain; HMG: high mobility group box.

Peripheral blood cells from patient X09, a 6-year old boy newly diagnosed with an immature CD4/CD8 double negative T-ALL, underwent whole-genome and RNA sequencing, revealing a translocation between chromosomes 5 and 11 and co-occurring mutations in *NRAS, NOTCH1, Krüppel-like factor 9 (KLF9), Cyclin-dependent kinase inhibitor 2A (CDKN2A), Sidekick cell adhesion molecule 1 (SDK1), T-cell receptor gamma (TRG),* and *transducin (beta)-like 1 X-linked receptor 1 (TBL1XR1)* (Fig. 1a). The t(5;11) translocation breakpoint occurred at *TCF7* intron 4–5 (Chr. 5) and *SPI1* intron 2–3 (Chr. 11) generating a predicted fusion transcript connecting exons 1–4 from *TCF7* to exons 3–5 of *SPI1* that was validated using both long-read nanopore and Sanger sequencing (Fig. 1b, c). At the protein level, the predicted fusion product brings together the first 182 amino acids from TCF1 (*TCF7*), including the N-terminal β-catenin binding region, and the terminal amino acids 52 to 271 of PU.1 (*SPI1*), resulting in a 402 amino acid long protein that still retains the DNA binding domain regions of PU.1 (Fig. 1d).

### Single-cell RNA sequencing identifies that high *SPI1* expression is associated with NRAS, stem cell, and Wnt/β-catenin signaling cell signatures

**Fig. 2:**
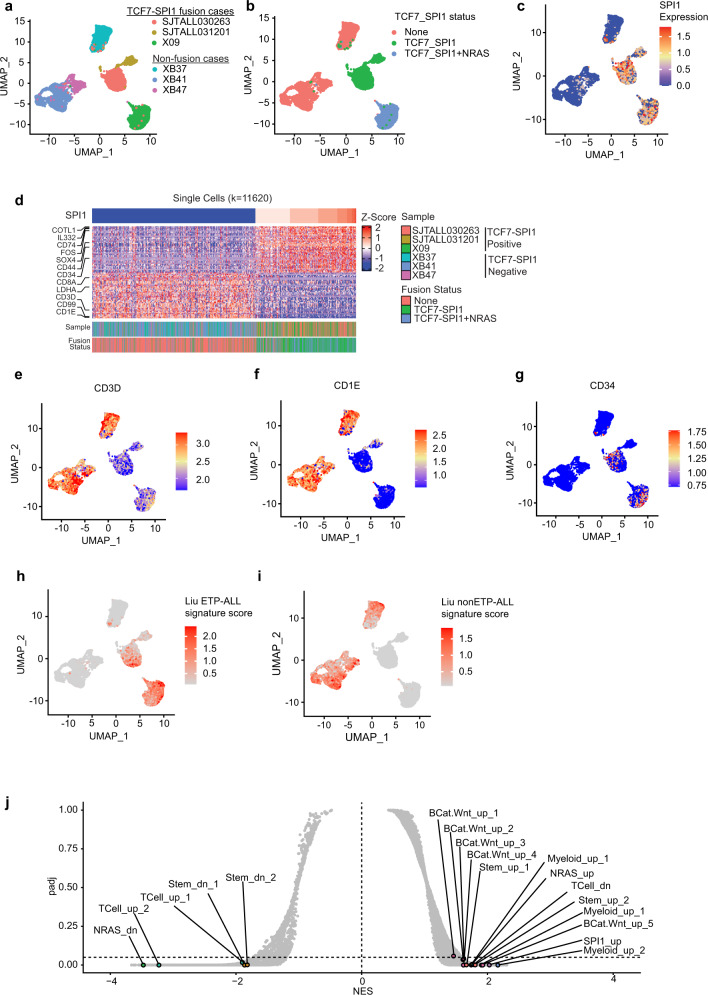
Single-cell RNA sequencing identifies that high *SPI1* expression is associated with NRAS, stem cell, and Wnt/β-catenin signaling cell signatures. **a**–**c** UMAP plot of 11,620 malignant T-cells from 6 T-ALL samples with cells colored by their patient of origin (a), TCF7-SPI1 and TCF7-SPI1 + NRAS mutation status (**b**), level of *SPI1* expression (**c**), the scalebar represents normalized expression values. **d** Heat map for all single cells across 6 T-ALL patients highlighting top 25 and bottom 25 genes correlated with SPI1 expression. **e**–**g** UMAP plot for expression levels of *CD3D* (**e**) *CD1E* (**f**) and, *CD34* (**g**) highlighting differential expression between fusion-positive and fusion-negative T-ALL cases**. h**, **i** UMAP plot of cells colored by the average expression of the ETP-ALL (**h**) or nonETP-ALL (**i**) signature derived from Liu et al.^5^, the scalebar represents averaged and scaled gene signature expression values. **j** Associated GSEA analysis pathways associated with *SPI1* expression highlighting enrichment of stem, Wnt/β-catenin, and myeloid signature. Full annotation and associated statistic of each pathway provided in Supplementary Data 8.

Single-cell RNA sequencing was carried out within six primary T-ALL samples (Supplementary Data 1–4) including patient X09 to identify transcriptomic determinants of this T-ALL subtype. Clustering a total of 13,848 cells from the pooled samples identified 22 cell clusters representing either malignant T cells or normal cells including NK-T cells, B cells, monocytes, and red blood cells (Supplementary Fig. 1a–c). Focusing on the 11,620 malignant T cells only, patients with *TCF7-SPI1* fusions were distinct from the other T-ALL samples and displayed significantly higher *SPI1* expression (Fig. 2a–c, Supplementary Fig. 1d). Since *SPI1* is the 3′ partner of the *TCF7-SPI1* fusion, and 10× short read RNA-sequencing is 3′ biased, we confirmed that *SPI1* expression levels were correlated with *TCF7-SPI1* fusion expression through long read nanopore sequencing (Supplementary Fig. 1e–h, Supplementary Data 5). Gene expression analysis also showed that SPI1 expression in fusion positive cases correlated with higher levels of hematopoietic stem markers like *CD34* and lower levels of mature T-cell markers like *CD3D* and *CD1E* (Fig. 2d–g) reconciling with the immature phenotype by flow cytometry analysis.

We next sought to determine how fusion positive cases compared to other defined T-ALL subtypes at the transcriptome level^5^. We found fusion cases clustered in close proximity to the early T- cell precursor (ETP) and immature Lim domain only 2/Lymphoblastic Leukemia Associated Hematopoiesis Regulator 1 (LMO2/LYL1) cases of the Liu et al. dataset (Supplementary Fig. 2a, b), suggesting fusion cases have a transcriptome similar to ETP-ALL despite their immunophenotype. Therefore, using published transcriptome data^5,25^, we next generated gene signatures from ETP- and nonETP-ALL patients (Supplementary Data 6, 7). The resulting Liu and Zhang ETP-ALL signatures were validated using single-cell RNA-sequencing data of normal developing human thymocytes^26^, confirming highest enrichment in cells within the designated ETP region. Conversely, the Liu nonETP-ALL and Zhang T-ALL signatures had the highest enrichment in T cells designated at double positive (DP) and αβT-entry regions (Supplementary Fig. 2c–k). Applying these signatures to our data, the three *TCF7-SPI1* fusion positive cases were positively enriched for an ETP-ALL signature and negatively enriched for the complementary T-ALL signature (Fig. 2h, i, Supplementary Fig. 3a–f).

Combining additional bulk RNA-sequencing data of these 6 patients with the cohort described by Seki et al. further revealed that patients harboring both the *TCF7-SPI1* fusion and *NRAS* mutations had a higher ETP-ALL signature compared to those with the *TCF7-SPI1* fusion alone (Supplementary Fig. 4). We also used GSEA analysis to determine signaling pathways associated with *SPI1* expression, which revealed a positive enrichment for myeloid, stem, and Wnt/β-catenin gene signatures (Fig. 2j, Supplementary Data 8). Collectively, these data highlight the fusion positive transcriptomes are enriched for an ETP signature and there is a strong link between *TCF7-SPI1* fusion status, RAS activation and the Wnt/β-catenin pathway.

### *TCF7-SPI1* requires mutant *NRAS* to cause in vitro transformation

**Fig. 3:**
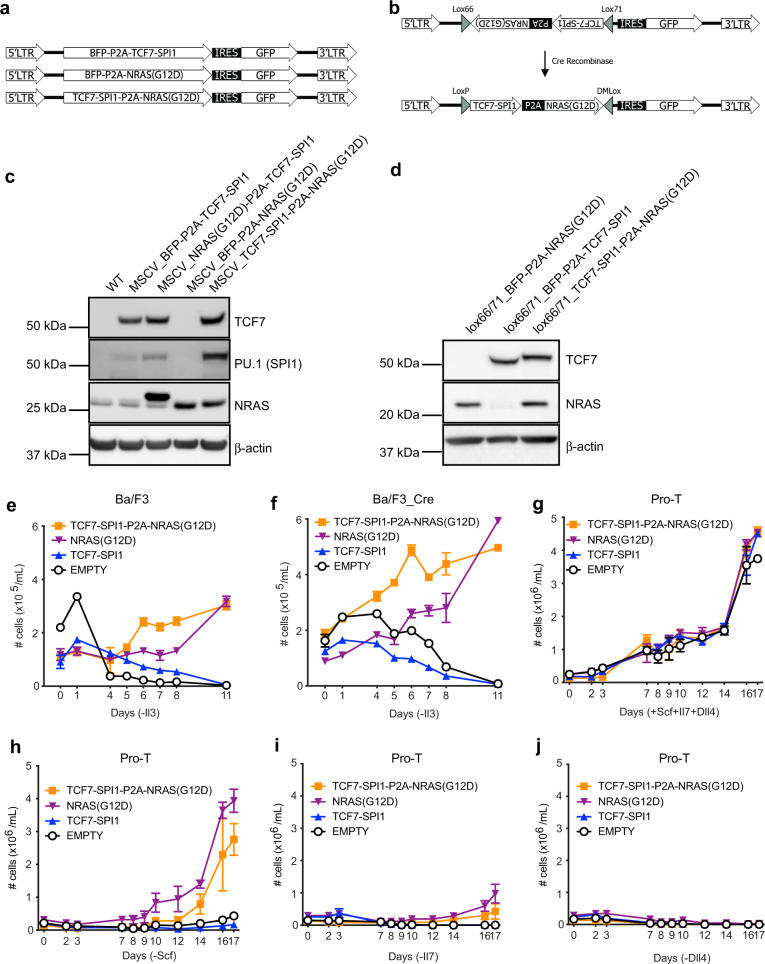
The presence of the *NRAS*(G12D) mutation is necessary for in vitro transformation to cytokine-independent growth. **a** Constitutive retroviral vectors with either BFP-P2A-TCF7SPI1, BFP-P2A-NRAS(G12D) or TCF7-SPI1-P2A-NRAS(G12D) followed by an IRES-sequence and GFP. **b** Schematic representation of the inducible Cre-Lox system. The same constructs as in (**a**) were cloned in the antisense direction between 2 anti-parallel asymmetric Lox66/Lox71 sites. Upon expression of Cre-recombinase the sequence is flipped in a unidirectional manner. **c**, **d** Western blot analysis showing expression of TCF7, SPI1, and NRAS for the different indicated constructs in Ba/F3 cells (**c**) (*n* = 1) or Cre-Ba/F3 cells with active Cre-recombinase (**d**) (*n* = 1). WT = Wild-type Ba/F3 and used as a control lysate. The viral P2A sequences within the expression vector results in a 3′ in-frame 18 amino acid sequence tail on the first protein expressed accounting for the increased molecular weight for NRAS(G12D) and TCF7-SPI1 in (**c**) and (**d**), respectively. **e** Growth curve of Ba/F3 cells transduced with the vectors illustrated in (**a**) or empty vector (white). **f** Growth curve with floxed constructs illustrated in (**b**) in Ba/F3-Cre cells alongside an empty vector control. **g**–**j** Growth curves in pro-T cells after transduction with the indicated constructs and empty vector. Either no growth factors (**g**), Scf (**h**), Il7 (**i**) or Dll4 (**j**) were omitted. **e**–**j** The number of cells is shown as a mean with standard deviation, *n* = 3 independent experiments for each condition.

A recent study identified recurrent translocations similar to our *TCF7-SPI1* fusion and these often harbored mutations in either *NRAS* (3/7 cases) or *KRAS* (1/7 cases)^9^. This same study showed that expression of *SPI1* fusions using an in vivo bone marrow transplant model led to a block in T-cell differentiation but not acute leukemia^9^. Given the high frequency of co-occurring mutations with *NRAS* (OR 5.833, *p* value 0.0067, Supplementary Data 9) and association with NRAS signaling (Fig. 2j) we sought to investigate whether the *TCF7-SPI1* fusion cooperates with oncogenic *NRAS* to drive T-ALL. We generated two sets of retroviral vectors that would express *TCF7-SPI1* and/or *NRAS*(G12D) from a single vector using a viral P2A sequence, in either a constitutive or inducible manner as has been previously described^6^ (Fig. 3a, b). Western blot analysis confirmed efficient constitutive or inducible expression of both the *TCF7-SPI1* (TCF1-PU.1) fusion and/or *NRAS*(G12D) upon transduction of Ba/F3 cells and Ba/F3-Cre cells respectively (Fig. 3c, d). The transformation potential was first determined in wild-type and Cre-positive Ba/F3 cells expressing *NRAS*(G12D) alone or in combination with *TCF7-SPI1*. Only when *NRAS*(G12D) was present, were the cells able to grow in an interleukin-3 (Il3)-independent manner, demonstrating that the NRAS(G12D) protein provides the essential proliferation and survival signals to lymphoid cells (Fig. 3e, f). Using a more physiological relevant Cre-positive pro-T cell culture model in combination with our inducible vector system, all transduced cells grew in the presence of Delta-like ligand 4 (Dll4), interleukin-7 (Il7) and stem cell factor (Scf). Notably, inducible expression of *NRAS*(G12D) + /− T*CF7-SPI1* provided growth and survival signals that could transform cells to Scf- and to a lesser extent Il7-independent growth, but all remained dependent on Notch signaling via Dll4 (Fig. 3g–j). Consequently, these results show that *NRAS*(G12D) alone is sufficient and necessary for transformation of these two in vitro cellular systems to cytokine-independent growth.

### *TCF7-SPI1* and *NRAS*(G12D) expression leads to aggressive T-cell leukemia in a mouse bone marrow transplant model

**Fig. 4:**
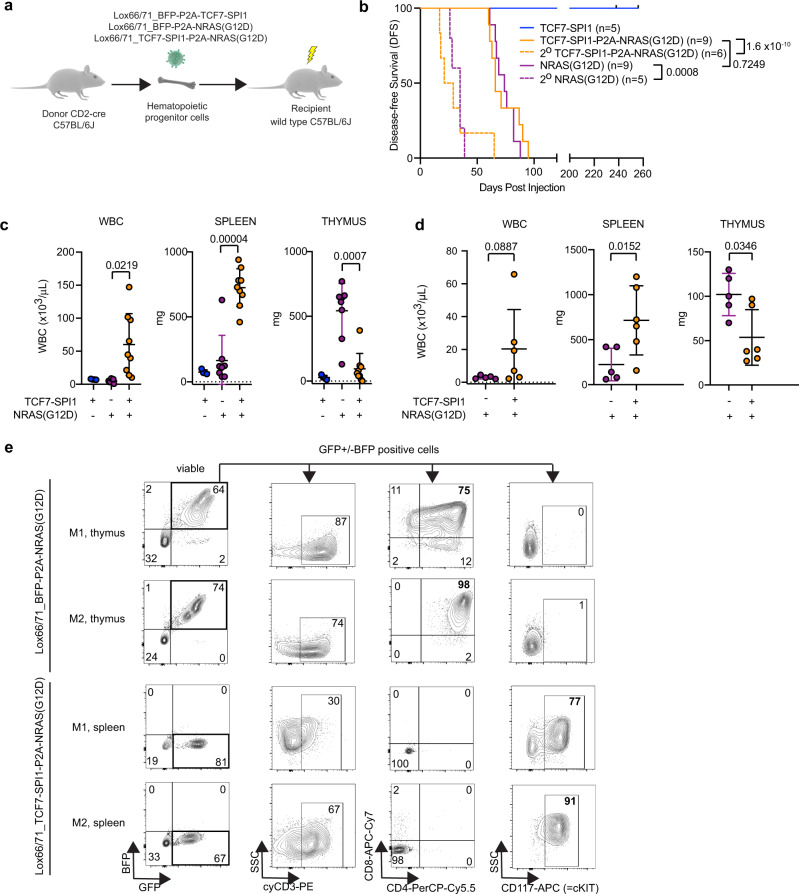
Conditional co-expression of TCF7-SPI1 fusion and NRAS(G12D) within developing T cells cooperate to generate an immature T-cell acute lymphoblastic leukemia. **a** Schematic representation of the conditional bone marrow transplant (BMT) model. **b** Survival curve showing the disease-free survival with no significant difference in disease latency between NRAS only and TCF7-SPI1-P2A-NRAS (*p* = 0.7429). Secondary transplantations (dashed lines) have significantly shorter disease latencies (*p* = 0.0008 for NRAS only; *p* = <0.0001 for TCF7-SPI1-P2A-NRAS). Log-rank (Mantel–Cox) test. **c** White blood cell (WBC), spleen weight and thymus weight at end stage for the primary BMT. *n* = 4 biologically independent animals for TCF7-SPI1, 8 for NRAS only (for the WBC graph *n* = 9) and *n* = 9 biologically independent animals for TCF7-SPI1 + NRAS in the three graphs. **d** Equivalent WBC, spleen, and thymus data for secondary transplantation. **c**, **d** Each point represents a different mouse, *n* = 5 biologically independent animals for NRAS only and 6 for TCF7-SPI1 + NRAS; the mean is shown with standard deviation. *P* values are indicated and were calculated by a one-way ANOVA with post hoc Dunnett T3 multiple comparisons test for (**c**) and a two-tailed Mann–Whitney test for (**d**). **e** Immunophenotyping of two different mice from (**b**) for each condition with spleen or thymus cells. Flow cytometry for cyCD3, CD4, CD8, CD117 are shown after gating on the viable and GFP+ cells. Distinguishing markers between the two conditions and their respective percentages are highlighted in red.

We next sought to determine whether the combination of *TCF7-SPI1* (TCF1-PU.1) and *NRAS*(G12D) cooperates in vivo to drive T-ALL. To this end, HSPCs isolated from the bone marrow of CD2-Cre positive C57BL/6J mice were retrovirally transduced with the different lox66/71 vectors (Fig. 3b–d) to restrict expression of *TCF7-SPI1* with or without *NRAS*(G12D) within developing T cells (Fig. 4a). Wild-type recipient mice that received *TCF7-SPI1* only transduced cells did not develop leukemia within the 200 days post injection observation period. Recipient mice that received *TCF7-SPI1* + *NRAS*(G12D) developed leukemia (median disease-free survival = 66 days) with a similar latency to those that received *NRAS*(G12D) alone (median disease-free survival = 74 days). Both were confirmed acute leukemias with secondary recipients succumbing to leukemia with a significantly decreased latency (Fig. 4b).

Although *NRAS*(G12D) alone and *TCF7-SPI1-P2A-NRAS*(G12D) both gave rise to leukemia with a similar latency, a clear difference was observed in disease presentation and immunophenotype. Mice expressing *NRAS*(G12D) alone within developing T cells had an enlarged thymus but rarely had increased white blood cell (WBC) counts or leukemic invasion of the spleen. In contrast, mice expressing both *NRAS*(G12D) and *TCF7-SPI1* presented with leukocytosis and splenomegaly but no enlarged thymus (Fig. 4c). This phenotype remained unchanged after secondary transplantation (Fig. 4d). The resulting leukemias were further phenotyped using flow cytometry with *NRAS*(G12D) leukemic thymic cells having a more differentiated CD4+/CD8+ immunophenotype and *TCF7-SPI1* + *NRAS*(G12D) leukemic cells a CD4−/CD8−, (intracellular) CD3+ and CD117+ immature immunophenotype (Fig. 4e). Subsequently, an in vivo limiting dilution assay was performed to evaluate the frequency of leukemic stem cells (LSCs). This showed a significantly higher LSC frequency in the *TCF7-SPI1-P2A-NRAS*(G12D) mice (1/1243) compared to the *NRAS*(G12D) only condition (1/312092) (Supplementary Fig. 5).

### *TCF7-SPI1*-induced immature leukemia requires upstream β-catenin activity

**Fig. 5:**
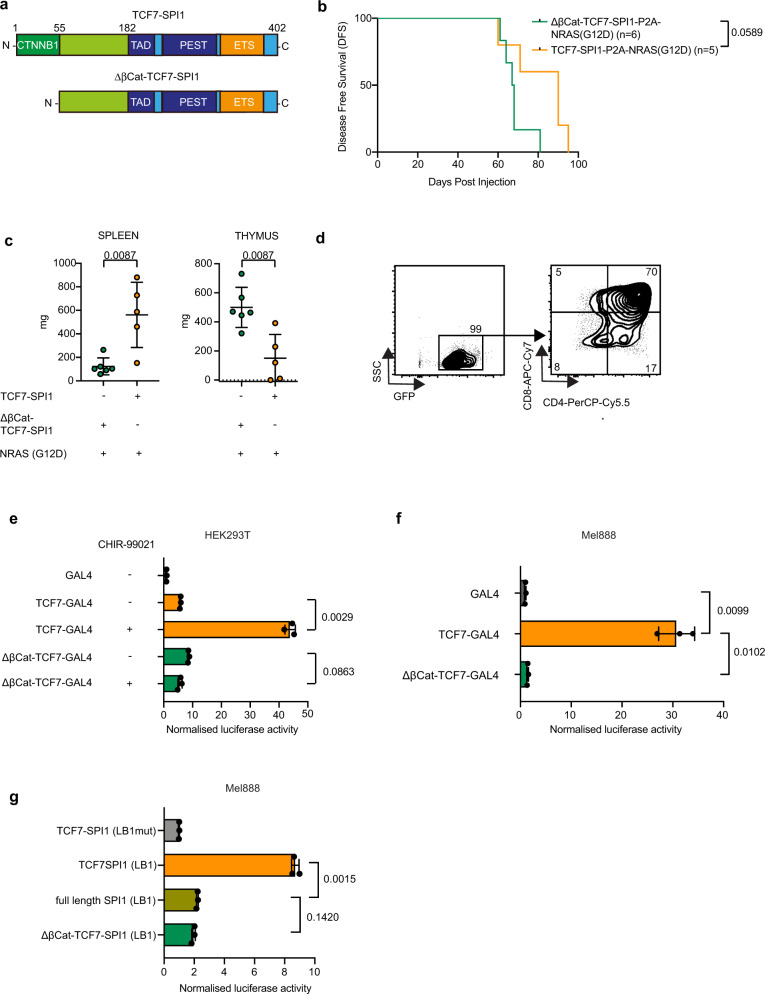
The N-terminal β-catenin binding site of the TCF1-PU.1 fusion protein is essential for transcriptional activity and ETP-ALL development in vivo. **a** Schematic representation of the protein domains resulting from the entire *TCF7-SPI1* fusion gene (above) or without (below) the β-catenin binding domain (CTNNB1) at the N-terminus (first 55 amino acids). **b** Kaplan–Meier curve for disease-free survival with the indicated inducible constructs. Log-rank (Mantel–Cox) test, *p* = 0.0589. **c** Spleen and thymus weights at end stage for mice in (**b**). Dots represent different mice (*n* = 6 biologically independent animals for ΔβCat-TCF7-SPI1 and 5 for TCF7-SPI1); the mean is shown with standard deviation. *P* values are indicated and were calculated by a two-tailed Mann–Whitney test. **d** Flow cytometry staining of a thymus sample of a ΔβCat-TCF7-SPI1-P2A-NRAS(G12D) mouse. Staining for CD4 and CD8 is shown after gating on GFP-positive cells. **e**–**g** Dual luciferase assay as described in “Methods”. **e** HEK293T cells with GAL4 constructs. Overnight treatment with CHIR-99021 at a dose of 1 μM. **f** Mel888 cells with GAL4 constructs. **g** Mel888 cells with SPI1 reporter gene containing the LB1 promotor or mutant. Results were normalized to the average GAL4 signal. *P* values (**e**–**g**) are indicated, one-way ANOVA with post hoc Dunnett T3 multiple comparisons test. Mean with standard deviation is shown. Dots represent different samples, *n* = 3 independent experiments per condition. Repeat experiments of (**e**) and (**f**) are shown in Supplementary Fig. 5.

Given the identification of Wnt/β-catenin signaling signatures in association with *SPI1* expression in the single-cell data and the presence of a β-catenin binding domain at the N-terminal region of the fusion protein, we next determined whether the N-terminal β-catenin binding domain of TCF1 within the fusion protein is also important in leukemia development. TCF proteins are well known to be effectors of canonical Wnt signaling together with β-catenin^27^. Furthermore, β-catenin has previously been suggested to be critical for LSC self-renewal and more recently linked to maintaining LSC stemness and *SPI1* expression^28–30^. We, therefore, set up a new bone marrow transplant where we selectively removed the first 55 amino acids of TCF1 *(TCF7)* that comprise the β-catenin binding site (Fig. 5a). Similar to the leukemia that develops in *NRAS*(G12D) only mice (Fig. 4), recipient mice that received ΔβCat-*TCF7-SPI1* only had an enlarged thymus, limited splenic invasion, and a more mature CD4+/CD8+ immunophenotype with similar disease latency (median DFS = 67.5 days) (Fig. 5b–d). This indicates that the deletion of the β-catenin binding domain abolished the action of the fusion leading to a NRAS(G12D) only disease.

To further determine whether loss of β-catenin binding was responsible for the altered disease presentation, we generated a series of GAL4 DNA binding domain fusion constructs, containing the N-terminal *TCF7* part of the *TCF7-SPI1* fusion or a truncated ΔβCat-*TCF7*, and performed luciferase reporter gene expression assays to assess transcriptional activity in HEK293T cells in the presence or absence of the GSK3β inhibitor CHIR-99021 to activate upstream Wnt signaling and increase β-catenin levels. The activation of Wnt signaling resulted in increased luciferase activity only for the full length but not for the ΔβCat-*TCF7* construct (Fig. 5e, Supplementary Fig. 6a) and this was phenocopied in the melanoma Mel888 cell line, where β-catenin is constitutively active due to a S37F mutation^31^ (Fig. 5f). We observed a similar difference between *TCF7-SPI1* and ΔβCat-*TCF7-SPI1* fusion constructs using Lambda B1 or Fes promotor luciferase reporter assays that both have endogenous SPI1 binding sites^32^ (Supplementary Fig. 6b). The luciferase activity induced by full length *SPI1* is lower than the activity induced by the fusion *TCF7-SPI1* and is at a similar level as for the ΔβCat-*TCF7-SPI1* fusion (Fig. 5g). Taken together, these data demonstrate that the *TCF7-SPI1* fusion induced immature T-ALL phenotype is driven in part by upstream β-catenin activity.

### Genetic and small molecule antagonism of the *TCF7-SPI1* fusion results in leukemia phenotype differentiation

**Fig. 6:**
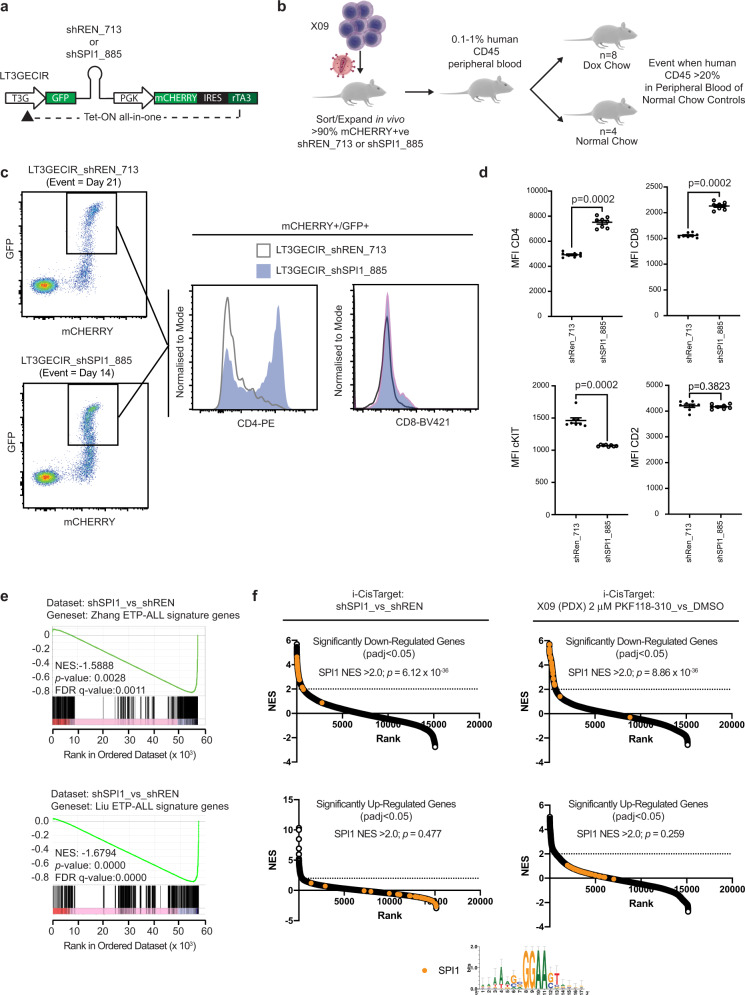
Genetic and small molecule antagonism of the *TCF7-SPI1* fusion results in leukemia phenotype differentiation. **a** Schematic representation of the shRNAmir in the LT3GECIR vector. **b** Schematic representation of the in vivo inducible knockdown mouse model. Mice received doxycycline or normal chow. **c** FACS plots for a shREN_713 (above) and a shSPI_885 mouse (below). Staining for CD4-PE (left) and CD8-BV421 (right) is shown after gating on GFP and mCHERRY positive cells from peripheral blood, shREN_713 is represented in white and shSPI_885 in purple. **d** Mean fluorescence intensity (MFI) of CD4, CD8, cKIT, and CD2 GFP+/mCHERRY+ peripheral blood is shown in mice with shREN_713 (*n* = 8) or shSPI_885 (*n* = 8). Dots represent different mice. *P* values are indicated and were analyzed with a two-tailed unpaired Mann–Whitney test. Mean is shown with standard error of the mean. **e** RNA-seq gene expression analysis in mice with shSPI1_885 versus shREN_713. GSEA using the Zhang and Liu ETP-ALL signature gene sets. Normalized enrichments scores, *p* values, and FDR *q* values are indicated. **f** iCisTarget motif analysis in mice with shSPI1_885 versus shREN_713 (left) or after 24 h ex vivo treatment with PKF 118-310 versus DMSO (right). Motifs in both down- (above) and upregulated genes (below) were ranked according to their respective normalized enrichment scores. SPI1 motifs are indicated in orange. *P* values are indicated and were calculated with a hypergeometric distribution. All results can be found in Supplementary Data 15–22.

To further characterize the function of the *TCF7-SPI1* fusion in leukemic maintenance and survival, we undertook an inducible knockdown strategy using an all in one Tet-On vector system to express short hairpin RNAs (Fig. 6a)^33^. X09 PDX cells were transduced with constructs encoding either a shRNAmir targeting the fusion transcript (shSPI1_885) or control Renilla luciferase (shREN_713), sorted and expanded to reach >90% mCHERRY positivity in vivo, then divided to receive either doxycycline or control chow at 0.1–1% human CD45 in the peripheral blood (Fig. 6b). At an end stage determined a priori, there was a 50% decrease in fusion expression but no decrease in leukemia burden in the peripheral blood or reduction of spleen weight upon doxycycline-induced knockdown of the fusion (Supplementary Fig. 7a, b). Strikingly, there was a significant increase in CD4 and CD8 and loss of CD117 (cKIT) cell surface expression with concomitant changes at the mRNA level (Fig. 6c, d, Supplementary Fig. 7b). This increased phenotypic differentiation was further supported by RNA-seq analysis, where knockdown of the fusion significantly decreased the ETP-ALL signatures (Fig. 6e, Supplementary Fig. 7c).

A global analysis of the differentially expressed genes to identify common regulatory elements using iCisTarget^34^ revealed that the significantly downregulated genes were highly enriched for SPI1 binding motif (Fig. 6f). To confirm the requirement of β-catenin for the oncogenic function of *TCF7-SPI1* and to confirm the effect of fusion knockdown on differentiation, we made use of the small molecule β-catenin/TCF antagonist PKF 118-310^27,35^. PKF 118-310 completely abrogated the luciferase signal generated by TCF7-GAL4 or TCF7-SPI1 in Mel888 cells phenocopying the genetic removal of the β-catenin binding domain (Supplementary Fig. 7d). Moreover, similar to the iCisTarget results after knockdown of the fusion, treatment with PKF-118-310 in three *TCF7-SPI1* fusion positive PDX samples also showed an enrichment of SPI1 motifs in the downregulated genes and decreased cKIT expression (Fig. 6f, Supplementary Fig. 7e, f). However, ex vivo treatment with PKF 118-310 did not show specific cytotoxicity toward fusion positive T-ALL cases compared to fusion-negative T-ALL cases (Supplementary Fig. 7g).

Taken together, these data demonstrate that the interaction of β-catenin with TCF1 (*TCF7*) is important for increasing the oncogenic function of the *TCF7-SPI1* fusion and that its inhibition leads to downregulation of SPI1 targets and phenotypic differentiation of the leukemic cells.

## Discussion

In 2017, Seki et al. described a distinct subset of pediatric T-ALL cases with dismal prognosis, characterized by an aberrant expression of *SPI1* fusion genes. Seven out of 181 investigated patients carried translocations encompassing the *SPI1* locus on chromosome 11 with *TCF7* or *STMN1* as typical fusion partners. Even though *TCF7* appeared to be a stronger activator of PU.1 than *STMN1*, the authors were unable to induce T-cell leukemia in a mouse model by constitutively expressing *TCF7-SPI1* on its own^9^. This suggests that additional proliferative and oncogenic signals are needed for leukemic transformation. In our center we identified a patient with a CD4/CD8 double negative T-ALL, characterized by the *TCF7-SPI1* fusion in combination with a *NRAS*(G12D) mutation. The frequency of mutations in RAS signaling in pediatric T-ALL is estimated to approximate 15%, but in the cohort of Seki et al., *NRAS* mutations were found in almost half of the cases with a *SPI1* fusion^9,36^.

In this current work, we were able to demonstrate that the *TCF7-SPI1* fusion can cooperate with *NRAS*(G12D) to induce an aggressive, CD4/CD8 double negative T-cell leukemia in vivo. Concordant with Seki et al., *TCF7-SPI1* alone did not induce T-ALL in our bone marrow transplant model. Expressing *NRAS*(G12D) alone using our conditional retroviral expression system limiting NRAS(G12D) to developing T cells caused a mature T-cell leukemia/lymphoma characterized by thymus enlargement. Other research groups have also reported on the development of hematologic diseases such as myeloproliferative diseases and T-ALL when expressing oncogenic RAS mutations in mouse progenitor cells^37,38^. However, combining both oncogenic alterations in our conditional bone marrow transplant model resulted in an aggressive acute T-ALL characterized by hyperleukocytosis, splenomegaly and the absence of thymus enlargement. Although no difference in disease latency was observed, the altered disease presentation when both TCF7-SPI1 and NRAS(G12D) are expressed was also found to cause a significantly higher frequency of leukemic stem cells. The combination also resulted in a more immature immunophenotype than mutated *NRAS* alone with a double negative leukemia (CD4− and CD8−), similar to the immunophenotype of the patient X09. This suggests that the *TCF7-SPI1* fusion provides a transcriptional program to block differentiation in the early thymic progenitor stage of development. Recently, it was shown that the *TCF7-SPI1* fusion blocks differentiation of murine T cells in the DN3a stage^39^.

This is also in part supported by our earlier findings^40^, where the order of mutational acquisition predicted that the translocation leading to the *TCF7-SPI1* fusion occurred prior to acquisition of the NRAS mutation. Hence, the observed immature T-ALL phenotype in our conditional bone marrow transplant models is the result of the fusion blocking T-cell differentiation and the *NRAS*(G12D) mutation providing the necessary proliferative signals for leukemia development. This model was also recently described for a *TCF3-HLF* fusion protein in B-ALL which cooperates with ETS factors and also leads to a block in differentiation^41^. Our data, therefore, posit that the observed co-occurrence of *SPI1* fusions with *RAS* mutants is driven by a positive selective pressure in the development of T-ALL.

We also demonstrated that the *TCF7-SPI1* fusion is dependent on β-catenin. Genetically, the *TCF7-SPI1* construct lacking the β-catenin binding domain was sufficient to alter the disease presentation in vivo to be equivalent to *NRAS*(G12D) alone with a more mature CD4+/CD8+ immunophenotype. This was further demonstrated using PKF 118-310, which specifically inhibits the interaction between β-catenin and TCF1 and led to significant downregulation of *SPI1* target genes in three separate *TCF7-SPI1* fusion PDX cases.

In normal T-cell development, the controlled expression of the *SPI1* transcription factor plays an important role in delaying the onset of T-cell commitment and possibly contributing to the expansion of early T cells^42^. In T-ALL, the mutually exclusive and ectopic expression of transcription factors such as TLX1, TLX3, and TAL1 is a key feature of T-ALL and often results from chromosomal translocations^43^. In this current work, we demonstrate that ectopic expression of *SPI1* can occur via chromosomal translocations and a unique situation where β-catenin hijacks the *SPI1* transcriptional program. This is in line with the recent finding that expression of *SPI1* is important for maintaining the ‘stemness’ of T-ALL leukemic stem cells in a regulatory loop involving both HAVCR2 and β-catenin^30^. Given these findings, we speculate that the ectopic and deregulated expression of *SPI1* may be a more general feature of immature T-ALL that either occurs by chromosomal translocations or potentially by cryptic mutations in cis-regulatory regions as has been recently described for TAL1^44^.

Targeting the fusion in vivo with an inducible knockdown system did not have a profound effect on leukemia burden. However, decreasing expression of the fusion did lead to increased differentiation, providing evidence the differentiation block observed in murine systems is directly relevant to clinical samples in maintaining leukemic cells in an immature state. Induction of fusion knockdown at an earlier time point before reaching 1% human CD45 in the peripheral blood might have resulted in an eventual exhaustion of T-ALL through differentiation even in the presence of the *NRAS* mutation but could not be answered in our in vivo experiment conditions where our ethical end point was predetermined. Of interest is the comparison with acute promyelocytic leukemia (APL), which was the paradigm of differentiation therapy. In recent years, it has become clearer that by targeting the *PML-RARα* fusion, other mechanisms, such as senescence, are also contributing to the clearance of APL cells^45^. In many other leukemias, e.g., IDH mutant AML, mutations can cause a differentiation block, but are dispensable for disease maintenance^46^. Here we show that such a therapy might be relevant for the first time in T-ALL upon the development of improved TCF-β-catenin interaction inhibitors that may also be able to eliminate leukemic stem cells. In those cases that also have *NRAS* mutation, combinatorial treatments might be required. Interestingly, during the revision of this manuscript, Gocho et al. identified T-ALL cases that are sensitive to dasatinib, some of which were positive for *TCF7-SPI1* without a NRAS mutation^39^. What remains unclear is the precise mechanism by which mutated *NRAS* and the *TCF7-SPI1* fusion cooperate to drive T-ALL. NRAS might exert its oncogenic function through the MAPK pathway. On the other hand, RAS is known to activate the PI3K/AKT pathway, thereby inhibiting GSK3β and thus activating β-catenin^47^. Independent of the mechanism, we do show for the first time that antagonism of the TCF/β-catenin interaction is sufficient to induce differentiation, providing a unique proof of principle that, in this instance, it is possible to directly target an oncogenic transcription factor.

In conclusion, we have shown that *TCF7-SPI1* cooperates with *NRAS* (G12D) to drive an aggressive immature T-cell leukemia in vivo and that it is possible to target this fusion protein by inhibiting protein-protein interactions between TCF1 and β-catenin. Patients with the *TCF7-SPI1* fusion could thus potentially benefit from an improved pharmacological targeting of the TCF/β-catenin interaction.

## Methods

### Patient sample collection and storage

Fresh bone marrow and peripheral blood samples were collected from newly diagnosed ALL patients in the Pediatric Hemato-Oncology Department of the University Hospitals Leuven at time of diagnosis and at remission on protocol S57176 approved by the Ethical Committee University Leuven. Written informed consent was obtained from every patient in accordance with the Declaration of Helsinki. Mononuclear cells were isolated using Ficoll-Paque and viably frozen in 95% fetal calf serum and 5% DMSO, or immediately injected into immune deficient NOD.Cg-*Prkdcscid Il2rgtm1Wjl*/SzJ (NSG) mice for further leukemic expansion.

### Whole-genome and transcriptome sequencing

DNA and RNA were extracted from the diagnostic sample (Maxwell, Promega) and prepared for sequencing using the KAPA Hyper Prep kit (Illumina). Libraries were sequenced on a HiSeq2500 with 125 bp paired-end reads (Illumina). Poor quality reads were trimmed using fastq-MCF (ea-utils)^9^ and the quality was checked with fastQC (v0.11.9) (http://www.bioinformatics.babraham.ac.uk/projects/fastqc) before alignment with Bowtie2 (v2.3.5.1) (DNA)^48^ or Tophat2 (v2.1.1) (RNA)^49^ to human reference genome GRCh37. Duplicates were removed using Picard and we applied VarScan (v2.4.4)^50^ to identify true somatic mutations and indels between the leukemia and remission samples. We included somatic mutations with a VAF of at least 5% in the tumor sample, in order to allow detection of mutations in minor subclones. In addition, the relative difference between the VAF in the tumor and the VAF in the remission sample was required to be minimum 20% and significant based on Fisher’s exact test, and the VAF at remission was <5%^40^. Chromosomal translocations were determined in the DNA by BreakDancer (v1.1_20100719) and we used FusionCatcher (v1.33) to detect gene fusions in the RNA data. Chromosomal breakpoints and mutations of interest were visually confirmed in IGV (v2.8.2) and verified by PCR and Sanger sequencing.

### Nanopore sequencing of primary X09 sample

Viably frozen diagnostic peripheral blood cells were thawed at 37 °C followed by suspension in phosphate-buffered saline (PBS) supplemented with 10% fetal calf serum. After the cells were washed, mRNA was extracted using the Fast track MAG – maxi mRNA isolation kit (Thermo Fisher Scientific) and prepared for sequencing on the GridION X5 (Oxford Nanopore Technologies) according to the manufacturer’s protocol. The sequencing data was then mapped to the human reference genome (hg38) using Minimap2 (v2.17-r941), after which it was inspected with the IGV (v2.8.2) software^51^.

### Flow cytometry

Cells were washed with PBS and prepared at a concentration of 1 × 10^6^/mL before staining and analysis on the MACSQuant VYB (Miltenyi Biotec), Fortessa (BD) or FACS Verse (BD). For the primary/xenograft and mouse cells, the antibodies listed in the supplementary table were used (Supplementary Data 10). FlowJo (v10.6.1; Tree Star) software was used to analyze the data. Full immunophenotypic profile of the patient samples can be found in Supplementary Data 1–3.

### Cell culture and retroviral transduction

293T cells were cultured in RPMI medium supplemented with 10% fetal bovine serum (FBS) and transfected using GeneJuice (Merck Millipore). Ba/F3 cells were cultured in RPMI medium with 10% FBS in the presence of 1 ng/mL interleukin-3 (Il3). Ba/F3-Cre cells were generated through retroviral transduction with MSCV-Cre recombinase-Puro vector and selection with 1 μg/mL puromycin (LifeTechnologies) addition in cell culture media. After transduction with retroviral vectors, cell transformation was assessed in the absence of Il3 growth factor. Pro-T cell cultures were established from C57BL/6 mice by extracting lineage negative bone marrow cells and growing them on DLL4-coated plates. Before coating with DLL4 (in-house production at VIB Protein Core), the non-treated tissue culture plates (BD Falcon #734-0949) are coated with anti-Fc (Abcam, ab1927). These cells are kept in culture in RPMI medium with 20% FBS, Il7 20 ng/mL (PeproTech), Scf 20 ng/mL (PeproTech) and Primocin 100 μg/mL (Invivogen). Cell transformation was assessed in the absence of the different growth factors^52,53^.

### Western blot analysis

Cells were lysed in cold lysis buffer, containing 5 mM NA_3_VO_4_ and Complete (Roche), before separation of the proteins on NuPAGE NOVEX Bis-Tris 4–12% gels (Invitrogen). Analysis was performed with antibodies against NRAS (Abcam, Ab55391), TCF1 (Thermo Fisher Scientific, MA5-14965), PU.1 (Abcam-76542), and β-actin (Sigma-Aldrich). Bands were visualized using ImageQuant LAS-4000 (GE Healthcare).

### Bone marrow transplant experiments

Lineage negative cells were isolated from the bone marrow of 6-to-8-weeks-old CD2-Cre C57BL/6J mice and transduced with retroviral vectors containing constructs encoding the *TCF7-SPI1* fusion and/or *NRAS* (G12D) mutant together with GFP downstream of an IRES. 0.5 to 1.5 × 10^6^ transduced cells were injected into the tail veins of irradiated C57BL/6 J female recipients. Blood samples were taken at regular time points and analyzed on a Vet ABC Hematology Analyzer (SCIL) and flow cytometer (MACSQuant® VYB, Miltenyi Biotec) to determine the evolution in white blood cell and GFP+ (BFP+) cell counts respectively. Mice were sacrificed when the white blood cell counts exceeded 20 000/µL and/or ethical end point criteria were reached. Animal experiments were approved by the Ethical committee on animal experimentation of KU Leuven.

### In vivo inducible knockdown mouse model

The LT3GECIR vector was provided by Prof. Dr. Johannes Zuber^33^ and shRNAmir sequences targeting Renilla Luciferase (Ren713) or human *SPI1* (SPI1_885) were cloned into EcoRI/XhoI sites (Supplementary Data 11). Lentivirus was generated in 293FT cells and then transduced into X09 patient derived xenograft (PDX) cells for 24 h prior to injection into NRG immunocompromised mice (NOD.Cg-Rag1tm1Mom Il2rgtm1Wjl/SzJArc) for expansion. Transduced cells were isolated from mice with mCHERRY positive cells sorted (FACS Aria III, BD) and enriched through multiple rounds of in vivo expansion until >90% of human CD45+ cells were mCHERRY+. One million mCHERRY positive transduced X09 PDX cells expressing either LT3GECIR_shREN_713 or LT3GECIR_shSPI1_885 were then inoculated into 12 NRG mice per group via tail vein injection. When mice reached 0.1–1% human CD45 in the peripheral blood, mice were randomly divided to receive either standard or Dox-impregnated chow (Doxycycline = 600 mg/kg; Specialty Feeds, Western Australia). Leukemia burden was monitored via weekly peripheral blood sampling until the mice reach a defined end point a priori of ~20% human CD45 positive cells in the blood. Authority to undertake animal work was approved by the Animal Care and Ethics Committee, University of New South Wales (Approval 20/6B)

### Limiting dilution assay

A secondary transplantation was performed with decreasing numbers of leukemic cells for both TCF7SPI1 + NRAS(G12D) and NRAS(G12D) only: 2 × 10^6^, 2 × 10^5^ cells, 2 × 10^4^ cells and 2 × 10^3^ cells. For each condition 5 WT C57BL/6 mice were injected after irradiation with 2,5 Gy, in total 40 mice were used. Disease was defined as a GFP percentage in the blood of 10%.

### Dual luciferase assay

HEK293T or Mel888 cells were transfected in a 12-well plate with 500 ng (3 μg for Mel888) of pGL 4.35 (Promega) and different GAL4 constructs (GAL4, TCF7-GAL4 and ΔβCat-TCF7-GAL4), and 25 ng of RL-TK (75 ng for Mel888). After 24 h the cells were lysed with Passive Lysis Buffer and light intensity was measured on a Victor multilabel plate reader (PerkinElmer) using the Promega Dual-Luciferase® Reporter Assay System (catalog number: E1910) according to the manufacturer’s instructions. For each well, firefly luciferase activity was normalized to Renilla activity. In the experiments with PKF 118-310 the results were normalized to either GAL4 or the mutant Lambda B1 reporter because of interference of PKF with Renilla luciferase activity. Previously, other drugs have also been reported to interfere with luciferase activity^54^. The used inhibitors were CHIR-99021 (Selleck Chem—S1263) and PKF 118-310 (Sigma-Aldrich—K4394). The Mel888 cells were kindly provided by the lab of prof. L. Larue. The SPI1 luciferase reporter genes were kindly provided by the lab of prof. O. Bernard.

### qPCR

Total RNA was extracted using using NuclepSpin RNA Plus columns (Macherey Nagel) and 500 ng reverse transcribed into cDNA using random primers according to the manufacturer’s protocol (GoScript cDNA Synthesis Kit, Promega). Real-time quantitative was performed using GoScript SYBR master mix kit (Promega) with the QuantStudio 3 PCR system.

(Applied Biosystem). Quality control, primer efficiency, and data analysis were carried out using qbase+ software (v3.2; Biogazelle). All gene expression was normalized using two housekeeping reference genes. The primer sequences for the qPCR experiments in Supplementary Fig. 7 can be found in Supplementary Data 12.

### Dose–response curve

Leukemic cells were seeded into a 96-well plate at 300,000 cells/mL and the drug was added using a D300e digital dispenser (Tecan) in an increasing dose. After 24 h, cell proliferation was measured using the ATPlite luminescence system on a Victor multilabel plate reader (PerkinElmer). Results were normalized to the DMSO condition. Results were analyzed with a non-linear regression. The analysis of the J188 PDX mouse was undertaken independently by the lab of Itaru Kato following the same protocol.

### Bulk RNA-seq analysis of patient cohorts

RNA-seq data were retrieved for 123 T-ALL patients from the National Bioscience Database Center (NBDC) of the Japan Science and Technology Agency (JST), under the accession JGAS00000000090^9^ and approved by the University of New South Wales institutional review board human ethics committee (HREAP HC200558). RNA-seq and patient-related data for two T-ALL patients (SJTALL03263, SJTALL031201) were obtained from St. Jude Hospital and approved by the University of New South Wales institutional review board human ethics committee (HREAP HC200562). Patients and/or their guardians for the samples SJTALL030263_D1 and SJT031201_D1 obtained from the St. Jude Biorepository, provided written informed consent in accordance with the Declaration of Helsinki. RNA-seq data previously generated in De Bie et al.^40^ for 4 T-ALL patients (X09, XB37, XB41, XB47) were also utilized. Paired-end reads were first trimmed to remove Illumina sequencing adapters and then filtered to remove poor quality or short read pairs (<20 base pair length, Phred33 quality score < 20) using TrimGalore (v0.6.6). Read pairs were then aligned to the human genome (GRCh38) using STAR (v.2.7.5c)^55^ in two-pass mode, whilst also retaining junction/chimeric reads for later fusion transcript detection. Aligned reads were then counted using HTSeq (v0.12.4)^56^ using GENCODE (v22) gene annotations. Gene-level read counts were additionally normalized to transcripts per million (TPM) using a custom R script (zenodo repository DOI: 10.5281/zenodo.4756105). Fusion transcripts were then detected using arriba (v1.2.0)^57^ with previously generated STAR outputs. Differential gene expression analyses were conducted between patient sub-groups using the DeSeq2 R package (v1.22.0)^58^, where differentially expressed genes were considered as those genes with a log_2_foldchange >1 or < −1 and Benjamini-Hochberg adjusted *p*-value < 0.05 (Supplementary Data 13). Gene set enrichment analyses (GSEA) were conducted using the fgsea R package (v1.14.0)^59^ against the molecular signatures database (v7.2), where input genes were ranked using -sign(log_2_foldchange)*log_10_(adjusted p-value). ETP-ALL and T-ALL gene signatures were derived from previous differential gene expression analyses in Zhang et al.^25^, where those genes that had fold change >2 and adjusted *p*-value < 0.05 were considered to be part of the ETP-ALL signature and those genes with fold change < −2 and adjusted *p*-value < 0.05 comprised the T-ALL signature (Supplementary Data 7 and 13). Gene signatures were calculated in each patient by first applying z-score scaling across signature genes, then taking the signature mean, and finally rescaling the signature means across all patients. ETP-ALL and nonETP-ALL gene signatures were also constructed by first performing differential gene expression analysis between samples with an ETP status of “ETP” (*n* = 19) and “nonETP” (*n* = 146) with the DeSeq2 R package(v1.22.0)^58^ using read counts deposited by Liu et al.^5^. Differentially expressed genes that had a fold change >2 and adjusted *p*-value <0.05 were considered to be part of the ETP-ALL signature and those genes with a fold change < −2 and adjusted *p*-value < 0.05 comprised the nonETP-ALL signature (Supplementary Data 6). Gene signatures for each patient were calculated as described above.

### Mutational analyses

Somatic mutations previously identified by targeted sequencing in Seki et al. were first obtained. Somatic mutations in the remaining 6 patients were derived from WGS data and annotated using the ensemble variant effect predictor (VEP) (Ensembl release 103)^60^. Only those mutations previously assessed in the targeted sequencing by Seki et al. were retained for association testing.

### Bulk RNA-seq for PKF 118-310 treated and inducible knockdown mouse samples

For differential gene expression analyses RNA was collected using NuclepSpin RNA Plus columns (Macherey Nagel) from triplicate conditions (2 μM of PKF 118-310 or DMSO treated PDX cells and sorted mCHERRY+GFP+ blasts isolated from the spleen for the Dox-inducible short hairpin RNA knockdown shREN_713 and shSPI1_885). The X09 PKF 118-310 treated RNA was prepared at the Genomics Core of KU Leuven and 3′ end RNA sequencing was performed (QuantSeq). The St. Jude SJTALL03263_D1 and SJTALL031201_D1 and inducible knockdown RNA samples were polyA-enriched and sequenced at BGI Genomics (Hong Kong) using DNBSEQ platform at a depth of 20 million read pairs per sample (PE100). After cleaning the data with fastq-mcf and a quality control with FastQC (v0.11.9), the resulting reads were mapped with HISAT2 (v2.1.0) to the human reference genome (GRCh38) and the abundance of reads per gene was determined with HTSeq-count. Differential gene expression was performed with the R package DESeq2 (v1.22.0),. Enrichment of motifs, based on a database of position weight matrices (PWMs), in the regulatory elements of the significantly up and downregulated genes (padj < 0.05) were determined with iCisTarget (v2015)^34^. Hockeystick plots based on the normalized enrichment scores (NES) were constructed, highlighting the PWMs related to SPI1. To determine the gene set enrichment of the ETP and T-ALL signature signatures in the ranked lists of differential genes the GSEA software of the Broad Institute was used.

### Single-cell RNA sequencing data analysis

Bone marrow samples from four T-ALL patients (X09, XB37, XB41, XB47) were previously profiled using the 10× Genomics Chromium^TM^ 3’v2 single-cell gene expression platform^40^. In addition, samples from two T-ALL patients (SJTALL030263, SJTALL031201) were sourced from the St. Jude Biorepository and profiled using 10x Genomics Chromium^TM^ 3’v3 chemistry. Reads were demultiplexed and aligned to the human reference genome (GRCh38), followed by unique molecular identifier (UMI) counting using the CellRanger v3.0.2 software suite^61^. Raw UMI count matrices were used as input for the Seuratv3 R package (v3.2.3) for downstream processing of the data^62,63^. In droplet-based scRNA-seq methods, droplets can often contain ambient RNA and be misinterpreted as a cell, therefore we eliminated these putative empty droplets using the DropletUtils package (v1.4.3), set at a false discovery rate (FDR) of 0.01 (1%)^64^. Furthermore, we filtered out “poor quality” cells that contained either; high proportions of ribosomal reads (>0.6), high proportions of mitochondrial reads (>0.1) or a low number of expressed genes (<200)^65^. All cells were then integrated and batch-corrected using sctransform whilst regressing out features in the data associated with technical variation, such as; the number of genes expressed, the number of UMI counts, mitochondrial read proportions, ribosomal read proportions, and estimated phases of the cell cycle^66^. In addition, the Harmony package (v1.0)^67^ was used to predict a shared embedding that accounted for any batch variation that arose due to the varying v2 and v3 10× Genomics Chromium^TM^ 3’ chemistry of samples. Harmony was used for initial cell type classification of malignant and non-malignant cell types but after removal of non-malignant cells, all embeddings were created using standard principal component analysis (pca) and uniform manifold approximation and projection (UMAP) based methods without Harmony. Principle components with the highest variance were used to semi-supervise cell clustering using the Louvain algorithm^62,63^, wherein the optimal clustering resolution was determined using the clustree R package(v0.4.1)^68^. Dimensional reduction was then performed using UMAP to visualize transcriptomic variation among single cells^69,70^. SPI1 expression correlations were computed using median-scaled log-transformed & normalized gene expression values from the “data” slot of the Seurat object. Correlation estimate was calculated according to the Pearson correlation coefficient. Expression heatmaps were generated for highly correlated and anti-correlated genes and visualized using the ComplexHeatmap package(v2.0.0)^71^. Gene-set enrichment analysis was undertaken on a dataset ranked according the SPI1 Pearson correlation coefficient, and enrichment statistics were evaluated for gene-sets of the Molecular Signatures database (MSigdb v7.4) using the fgsea package (v1.14.0)^59,72^. Gene signature expression scores of Zhang et al.^25^ and Liu et al.^5^ signatures were computed for each cell using the AddModuleScore function of the Seuratv3 R package^62,63^ and scores were then re-scaled using z-score normalization. Processed single-cell RNA-seq data of the human fetal thymic cell atlas were directly retrieved from the zenodo data repository under the accession 10.5281/zenodo.3572422. These data contained normalized count matrices, embeddings, and cell type annotations made in the original study^26^. Cells corresponding to either “T” or “innate T” cell types in “level_1” meta data annotations as well as early thymic progenitor (ETP) cells were subsetted. Gene signature expression scores were computed as described above. Two-sided pairwise *t* tests were performed between cell groups, followed by Benjamini-Hochberg (BH) adjustment of resultant p values to correct for multiple hypothesis testing.

### Amplification of the TCF7-SPI1 fusion from 10x cDNA libraries

A total of 0.1 ng of cDNA from 10x cDNA libraries for SJTALL031201_D1 was amplified using 2× KAPA HiFi HotStart ReadyMix and 0.4 μM Biotinylated TCF7 Exon 4 forward primer and 0.4 μM Read 1 reverse primer (Supplementary Data 14) using 1 cycle of initial denaturation 95 °C for 5 min, followed by 15 cycles of 98 °C denaturation for 20 s, 63 °C annealing for 30 s, 72 °C extension for 2 min; and then a final 72 °C extension for 5 min. PCR reactions underwent a 0.9X SPRIselect single sided size selection purification and eluted with 20 μL of nuclease-free water. Biotinylated PCR products were then captured using M-280 Streptavidin Dynabeads™ and washed 3× with 10 mM Tris-HCl (pH 7.5) 1 mM EDTA 2 M NaCl wash buffer prior to final resuspension in 60 μL nuclease free water. A second nested PCR was then carried out using 5 μL of the streptavidin bead resuspension using 2× KAPA HiFi HotStart ReadyMix and 0.4 μM Nested TCF7 Exon 4 forward primer and 0.4 μM Read 1 reverse primer using 1 cycle of initial denaturation 95 °C for 5 min, followed by 30 cycles of 98 °C denaturation for 20 s, 63 °C annealing for 30 s, 72 °C extension for 2 min; and then a final 72 °C extension for 5 min. The resulting nested PCR underwent a 0.9× SPRIselect single sided size selection purification and eluted with 20 μL of nuclease free water. Amplification was confirmed by TapeStation (Agilent) analysis and Sanger Sequencing (Supplementary Fig. 3) prior to nanopore sequencing.

### Nanopore sequencing and analysis

Amplified cDNA libraries were prepared for long-read sequencing using Oxford Nanopore Technologies (ONT) 1D adapter ligation sequencing kit (SQK-LSK109). Three samples were barcoded, pooled and sequenced on a single R9.4.1 PromethION flow cell (FLO-PRO002). MinKNOW live base-calling was performed using Guppy (v4.0.11). The resulting base-called FASTQ files were de-multiplexed by 10× cell barcodes, using a direct sequence matching strategy described previously^73^. Briefly, forward and reverse-complemented cellbarcode sequences (16 nt) were used to demultiplex the nanopore sequencing reads by scanning the first and last 200 nt of any read longer than 250 nt for a matching sequence, with <2 mismatches. Demultiplexed reads were then grouped into separate FASTQ files that were separately assembled de novo using Canu^74^ (version 1.8; parameters: -Overlapper = minimap -minReadLength = 500 -minOverlapLength = 100 -genomeSize = 1k -nanopore-raw -stopOnReadQuality = false) Demuxed FASTQ reads were then aligned to their respective Canu contigs using Minimap2^51^ (version 2.17-r943-dirty) before ‘polishing’ the consensus sequence using Racon^75^ (version 1.3.3; parameters: -u -w 200 -m 8 -x -1 -g -4). The Minimap2/Racon step was repeated a total of four times. The number of total nanopore reads and demultiplexed reads for each sample is shown in Supplementary Data 5. Reads spanning TCF7 exon #4, the fusion breakpoint and SPI exon #3 were classified as ‘fusion reads’, while reads spanning TCF7 exon #4 and spanning TCF7 exon #7 were classified as “canonical reads”. Canonical/fusion read counts are summarized in Supplementary Data 5.

### Statistical analysis

Statistical analyses were performed using the Prism software (Graphpad v9.0.2 (134)). Kaplan–Meier curves were used for the survival of mouse BMT’s with two-sided p values determined by log-rank (Mantel–Cox) test. For the comparison of 2 groups, an unpaired *t* test with Welch’s correction or Mann–Whitney test was used. Data in the luciferase experiments are expressed as means ± standard deviation (SD). Groups were compared using the post hoc Dunnett T3 multiple comparisons test after Brown–Forsythe ANOVA, *p* values are adjusted *p* values. Expression analysis for different subsets of patients was done with a Tukey’s multiple comparisons test. For the analysis of the dilution assay we used the ELDA (extreme limiting dilution analysis) software^76^.

### Reporting summary

Further information on research design is available in the Nature Research Reporting Summary linked to this article.

## Supplementary information

Supplementary Information

Description of Additional Supplementary Files

Supplementary Data 1-22

Reporting summary

## Data Availability

The data of bulk and single-cell RNA sequencing of 4 T-ALL patients (X09, XB37, XB41, and XB47), nanopore sequencing of X09, together with the single-cell RNA sequencing data of a PDX model of the X09 patient and sequencing data of two patients from the St-Jude cohort (SJTALL030263 and SJTALL031201), have been deposited within the European Genome-phenome Archive (EGA), which is hosted by the EBI and the CRG. Accession number EGAS00001005097, https://ega-archive.org/datasets/EGAD00001007010. The Seki et al. RNA-seq data are available at the National Bioscience Database Center, Japan Science and Technology Agency (ID: JGAD00000000090). The whole-genome sequencing data of the four T-ALL samples (X09, XB37, XB41, and XB47) from De Bie et al.^40^ can be accessed through the following link: https://ega-archive.org/datasets/EGAD00001003951. For restricted data, access can be requested by sending an email to Sofie Demeyer (sofie.demeyer@kuleuven.be) with requests for data use outside academic research, requiring a data access agreement. Processed single-cell data have been deposited with the Zenodo Biorepository (DOI:10.5281/zenodo.4756105, https://zenodo.org/record/4756105#.YMbypC0RqqB). For Gene Set Enrichment Analysis, Molecular Signatures Database was used (http://www.gsea-msigdb.org/gsea/msigdb/index.jsp). Source data are provided with this paper.

## Source data

Source data

## Code availability

Custom R-scripts used have been deposited with the Zenodo Biorepository (DOI:10.5281/zenodo.4756105).
