# Supplementary material for: Oncogenic cooperation between TCF7-SPI1 and NRAS(G12D) requires β-catenin activity to drive T-cell acute lymphoblastic leukemia
Source: Nat Commun. 2021 Jul 6;12:4164. doi: 10.1038/s41467-021-24442-9 (PMC8260768; doi:10.1038/s41467-021-24442-9)
Supplement: Supplementary file 1 — Supplementary Information [file 41467_2021_24442_MOESM1_ESM.pdf]

Supplementary Fig. 1

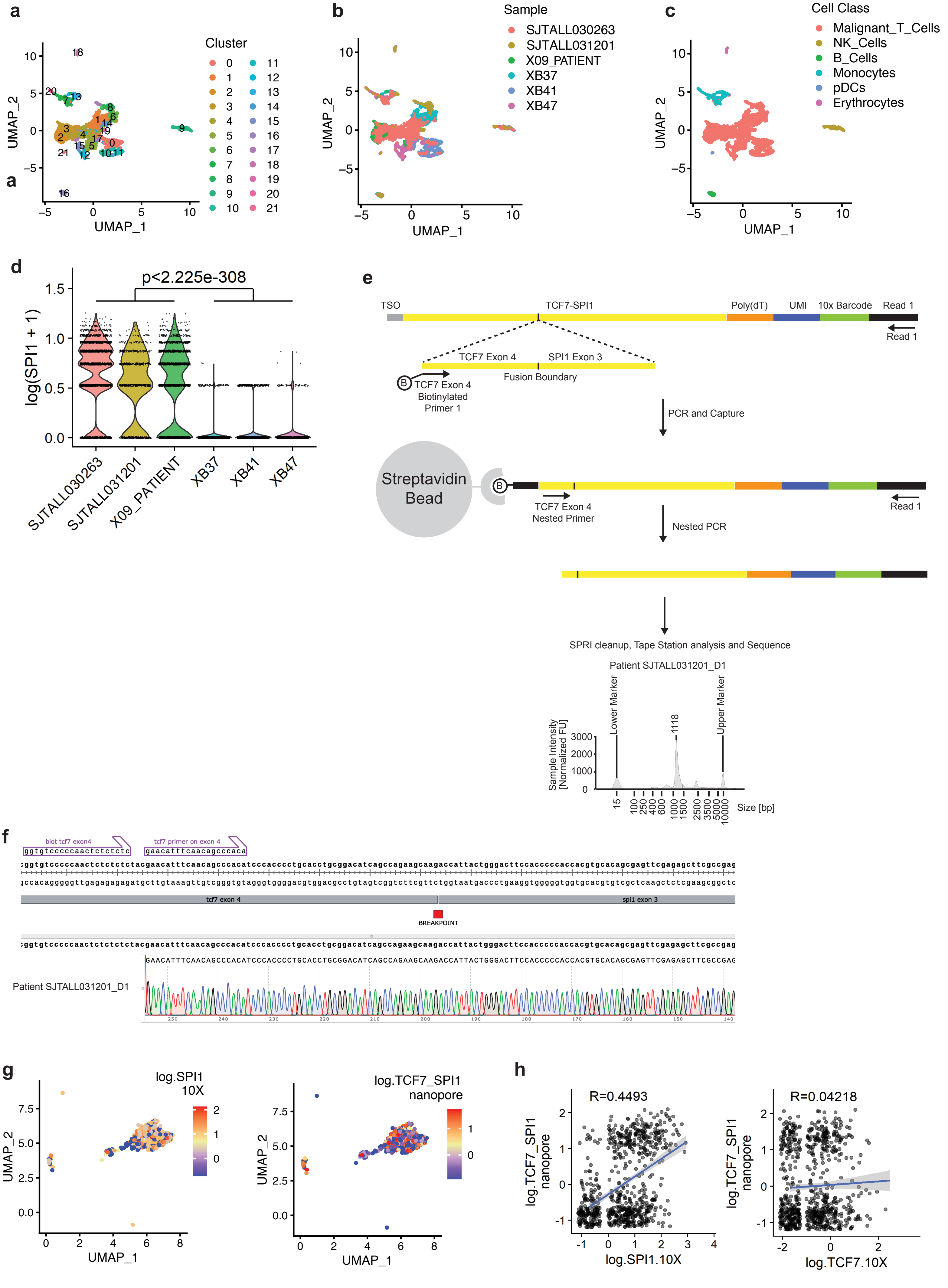

Supplementary Fig. 1: Single-cell analysis of 6 T-ALL patients.

a-c UMAP plot of 13848 cells from 6 T-ALL samples, cells are colored by cluster (a), sample of origin (b) and cell type classification (c). **d** Violin plots of *SPI1* expression across the 6 T-ALL sets. (two-sided t test comparing all cells from *TCF7-SPI1* fusion positive tumors vs *TCF7-SPI1* fusion negative tumors). **e** Schematic representation of PCR and Nest PCR strategy used to amplify *TCF7-SPI1* fusion transcripts to identify the 10x cellular barcode and the resulting TapeStation fragment analysis of the resulting PCR product highlighting a major peak at 1118 bp for SJTALL031201_D1. **f** Sanger sequencing result illustrating alignment with fusion reference sequences and the break point. **g** UMAP of patient SJTALL031201 for normalized *SPI1* UMI reads generated by the 10x library and adjacent normalized fusion UMI read counts from the nanopore sequencing. UMI counts were normalized in each cell by dividing total detected UMI across all aligned genes in each cell (from the 10x library) and then log1p-transformed. **h** Correlation of the normalized UMI counts from nanopore sequencing that amplified both *TCF7-SPI1* and *TCF7* transcripts (**Supplementary Data 5**) with normalized 10x UMI read counts for *SPI1* and *TCF7*.

Supplementary Fig. 2

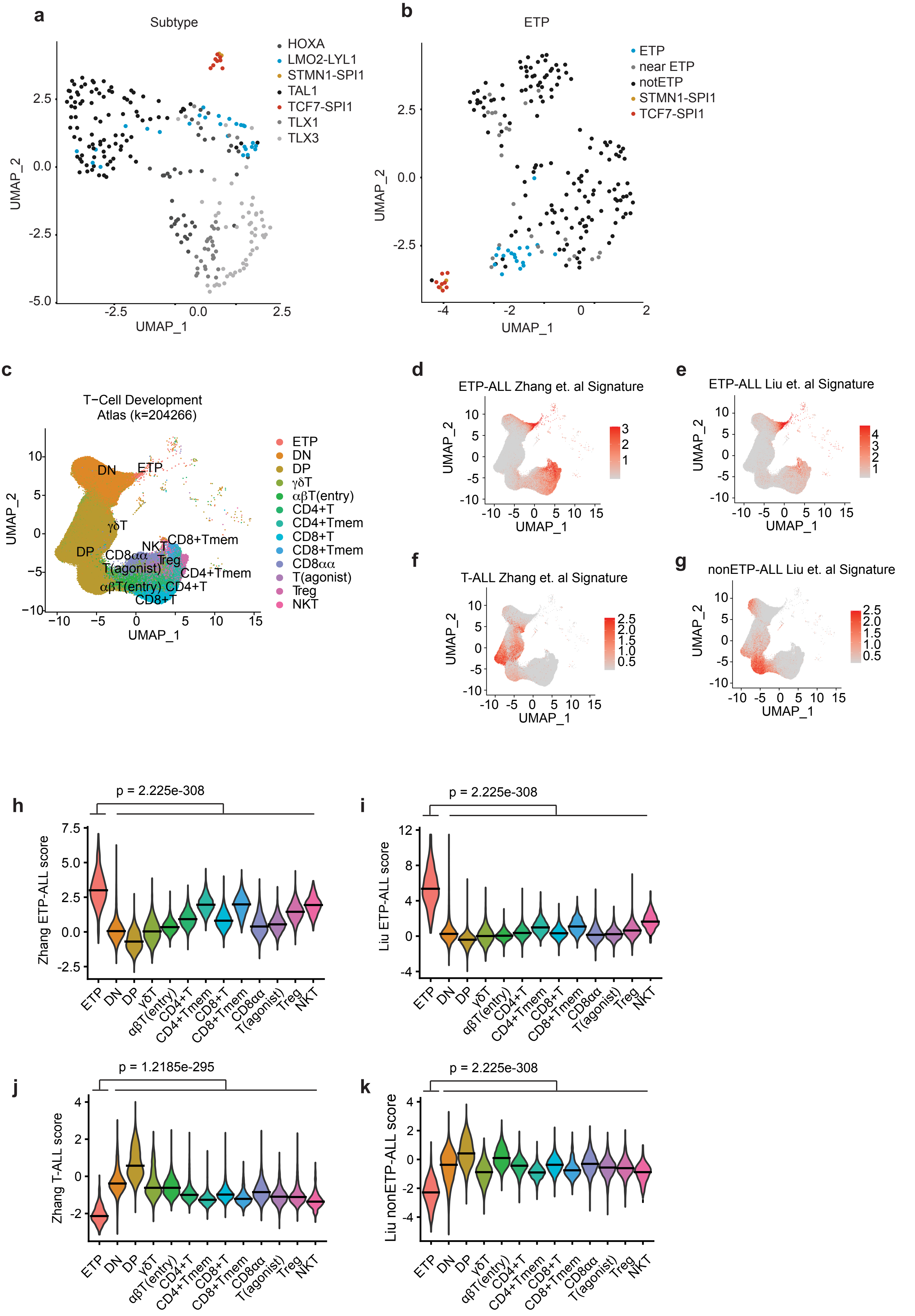

Supplementary Fig. 2. Single-cell analysis of fusion cases with respect to defined T-ALL subtypes and validation of ETP and nonETP-ALL gene signatures in a T-cell development atlas. a-b UMAP plot of cells of fusion cases with respect to defined subtypes of T-ALL and ETP status according to Liu et al.[1] **c-g** T-cell development atlas derived from Park et al.[2] (c) with corresponding enrichment of the Zhang ETP-ALL (d), Liu ETP-ALL (e), Zhang T-ALL (f) and Liu nonETP-ALL (g) signatures. The scalebar represents averaged and scaled gene signature expression values. **h-k** Corresponding violin plots for relative expression of the Zhang ETP-ALL (h), Liu ETP-ALL (i), Zhang T-ALL (j), and Liu nonETP-ALL (k) signatures. Two-sided unpaired student's t tests were performed to derive p-values between ETP cells and all other cells. Only one comparison was made, therefore no adjustment to the resultant p value was required.

Supplementary Fig. 3

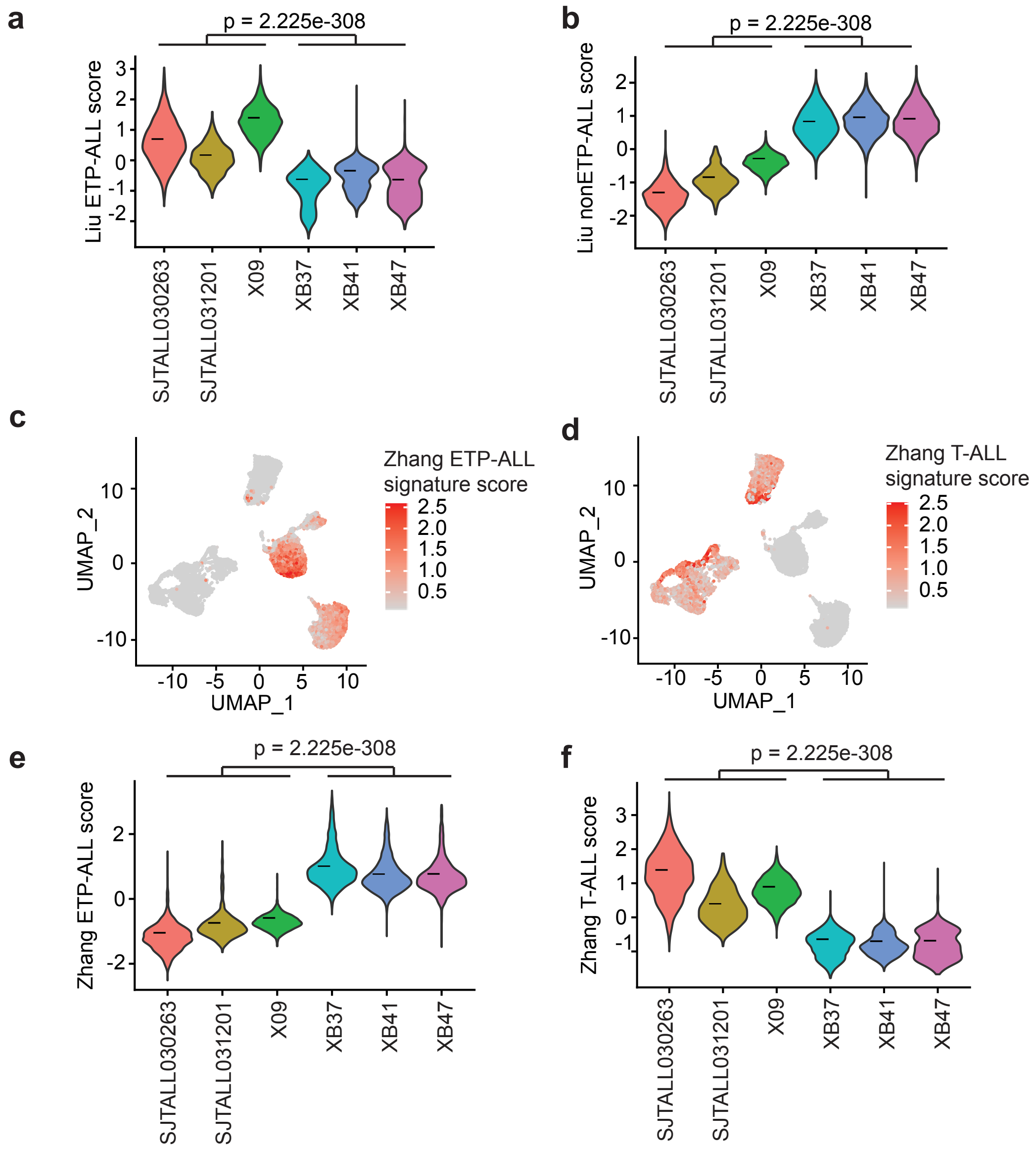

Supplementary Fig. 3: ETP signature enrichment plots on single-cell analysis of 6 T-ALL patients. a-b violin plots for Liu et al.[1] gene signatures scores corresponding to Fig. 2h-i**. c-f** UMAP plot of cells colored by the average expression of an ETP-ALL (c) or T-ALL (d) signature derived from Zhang et al.[3], the scalebar represents averaged and scaled gene signature expression values with corresponding violin plot for relative expression (e and f) below each UMAP. (a-b; e-f) Two-sided unpaired student's t tests were performed to derive p values between cells from *TCF7-SPI1* samples (SJTALL030263, SJTALL031201, X09) and all other samples (XB37, XB41, XB47). Only one comparison was made, therefore no adjustment to the resultant p value was required.

Supplementary Fig. 4

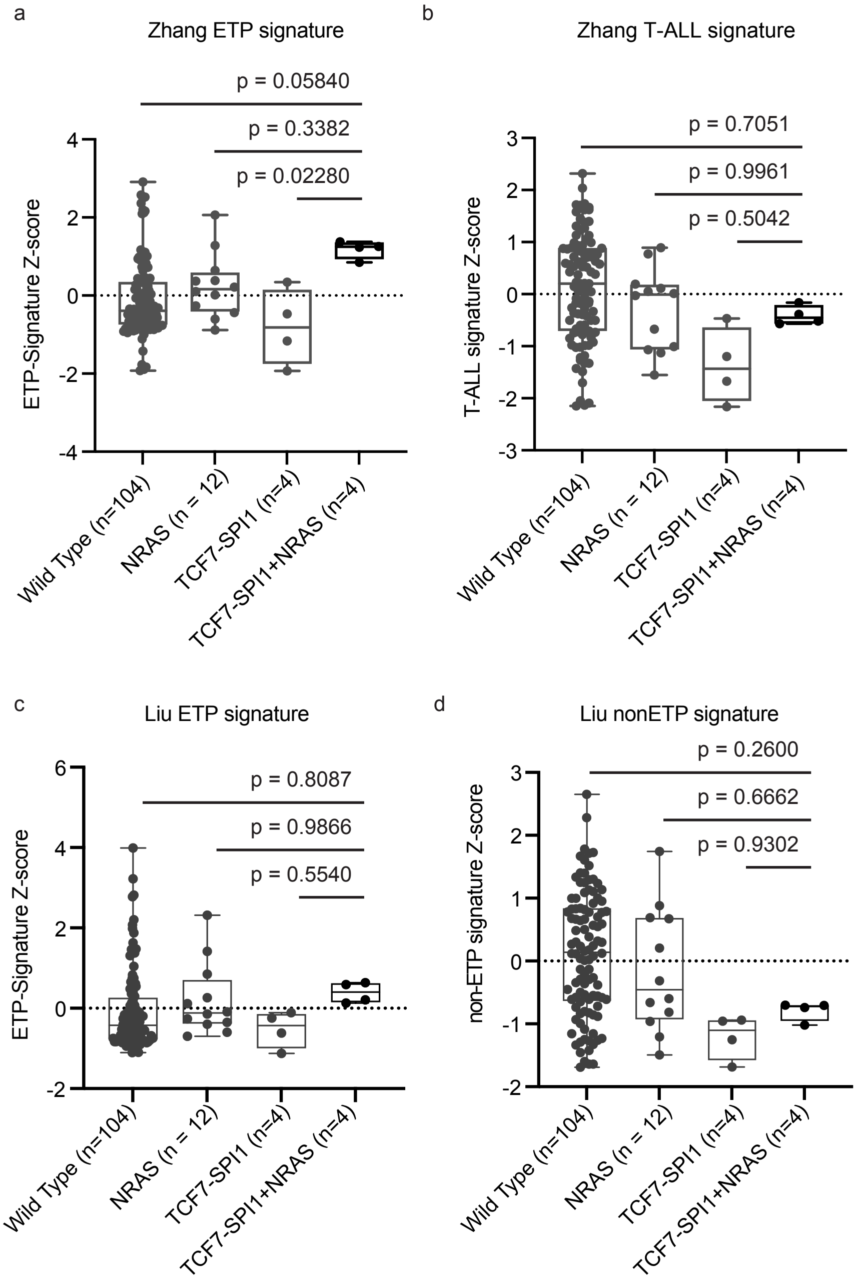

Supplementary Fig. 4: Expression of ETP-ALL and T-ALL signatures in TCF7-SPI1 cases with NRAS. **a-d** Expression for subsets of T-ALL patients with Zhang ETP-ALL (a) Zhang T-ALL (b), Liu ETP-ALL (c) and Liu nonETP-ALL (d) signatures. One-way ANOVA with Tukey's test for multiple comparisons. For each boxplot, the line in the center of the box represents the median expression value, and the lower/upper bounds of the boxes represent the interquartile range of all expression values (25th and 75th percentile of all expression values). The whiskers are drawn from the minimum and maximum expression values to the lower/upper bounds of the box. n = 104 (wild-type), 12 (NRAS), 4 (TCF7-SPI1) and 4 (TCF7-SPI1+NRAS) biologically independent samples.

Supplementary Fig. 5

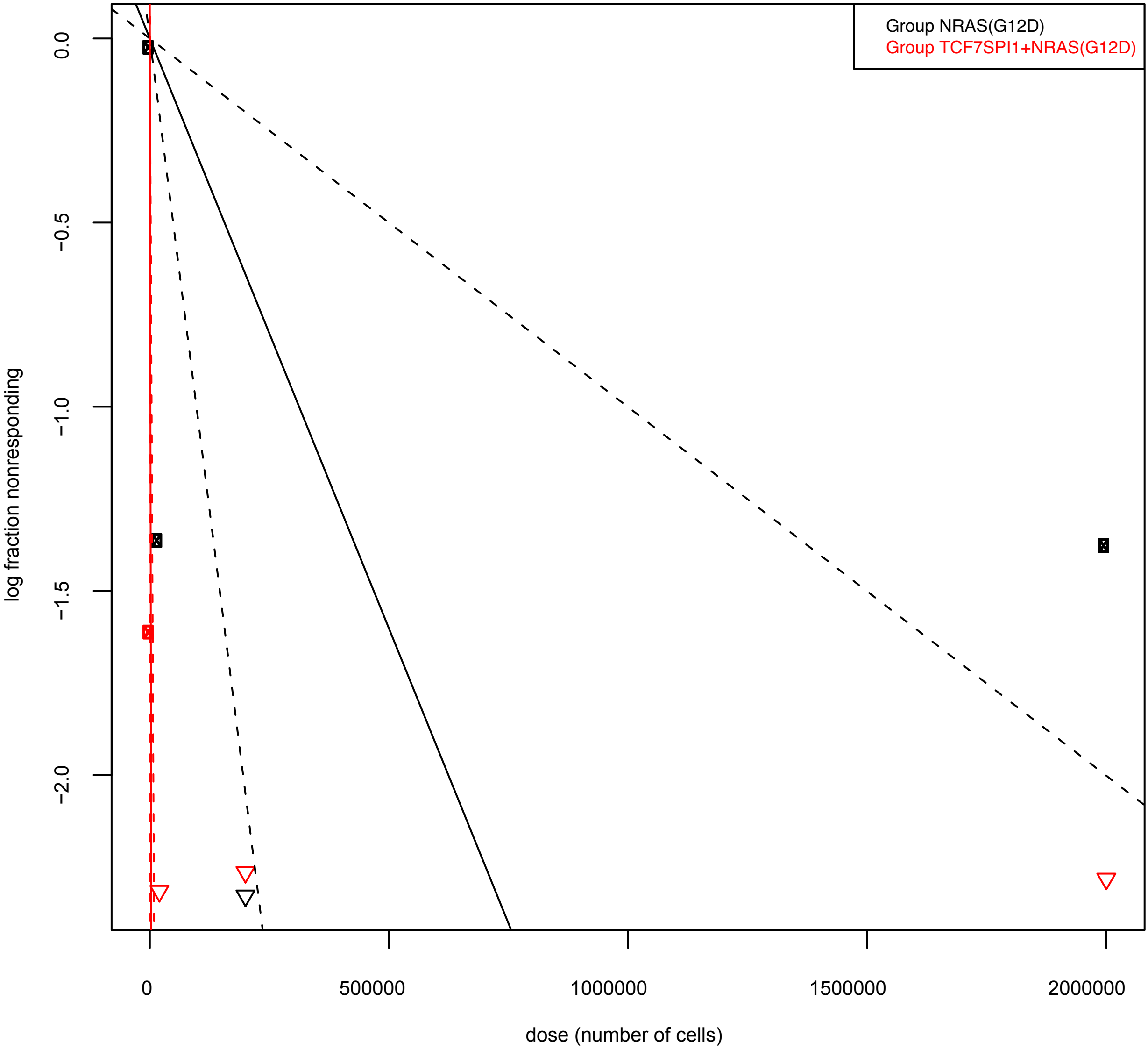

Supplementary Fig. 5: ***In vivo*** **limiting dilution assay for leukemic stem cell frequency.**

ELDA software plot showing the difference in leukemic stem cell frequency, 1/1243 for *TCF7SPI1+NRAS*(G12D) vs. 1/312092 for *NRAS*(G12D) only. Chi-squared test, $p = 2 \times 10^{-14}$.

Supplementary Fig. 6

a

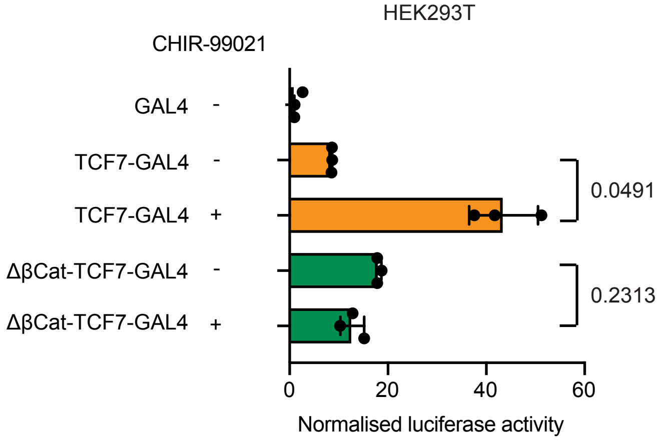

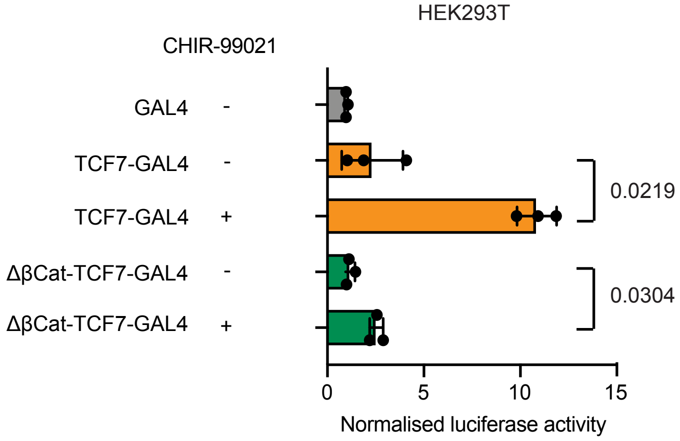

b

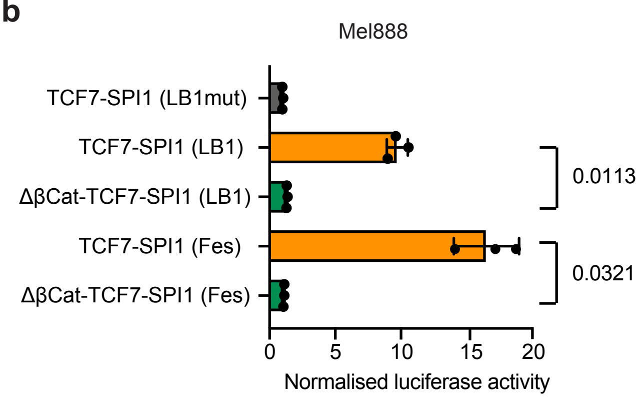

Supplementary Fig. 6: Luciferase experiments. a Repeats of the experiment in Fig. 5e. **b** Mel888 cells. Cells were transfected with a SPI1 reporter gene containing the Lambda B1 (LB1) promotor or Fes promotor and measured after 24h. (a-b) Mean with standard deviation is shown. Dots represent different samples, n = 3 independent experiments per condition. One-way ANOVA with post hoc Dunnett T3 multiple comparisons test.

Supplementary Fig. 7

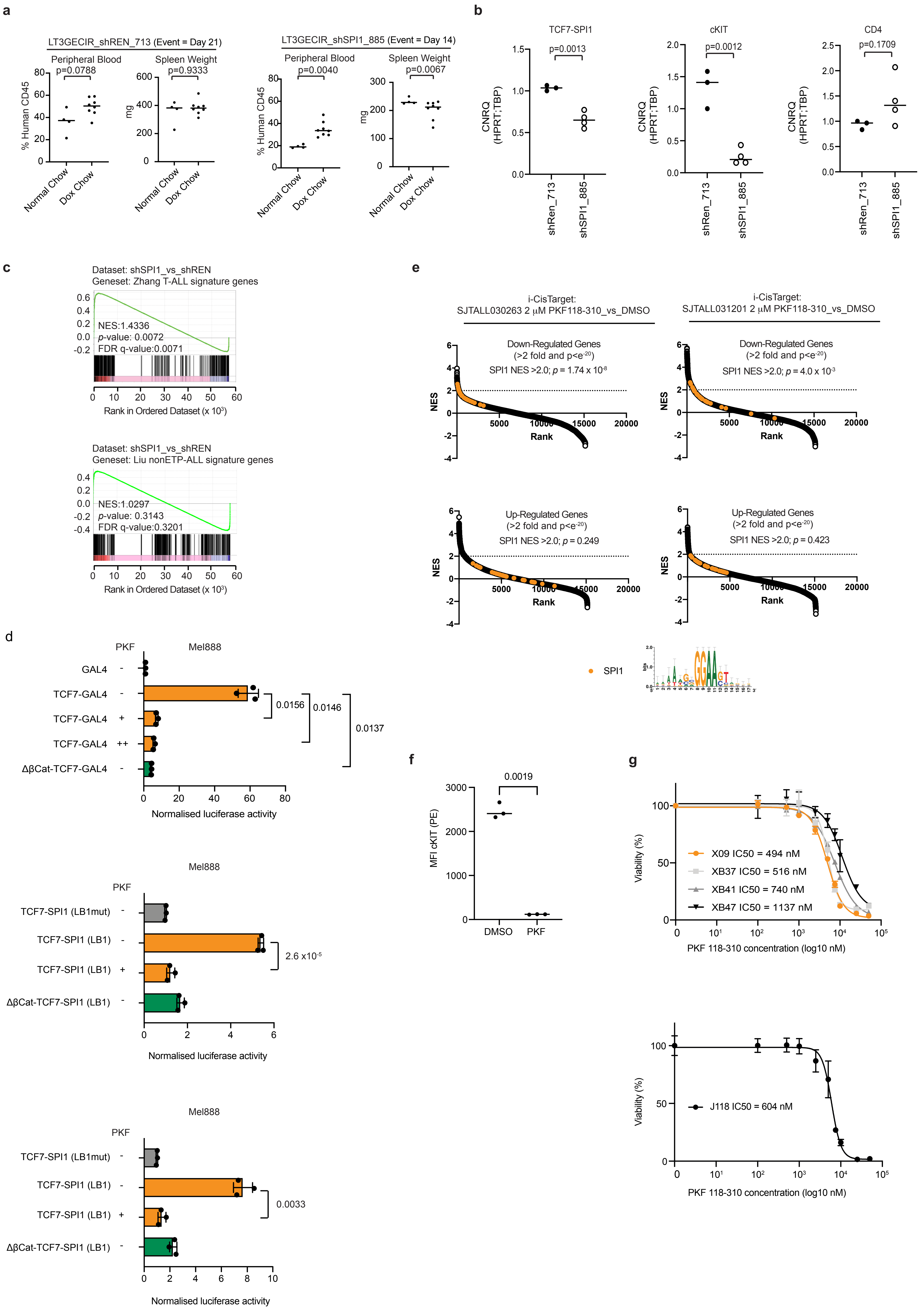

Supplementary Fig. 7: Treatment experiments. a Leukemia burden measured by FACS for human CD45 in the peripheral blood (left) or by spleen weight (right) at sacrifice. P values are indicated and were calculated by a two-tailed unpaired Mann-Whitney test. **b** qPCR results for *TCF7-SPI1*, *cKIT* and *CD4* expression after fusion knockdown with shREN_713 or shSPI1_885. P values are indicated and were calculated by a two-tailed unpaired t test. **c** RNA-seq gene expression analysis in mice with shSPI1_885 versus shREN_713. GSEA using the Zhang T-ALL and Liu nonETP-ALL signature gene sets. Normalized enrichments scores, p values and FDR q values are indicated. **d** Dual Luciferase Assay as described in Methods. Mel888 cells were treated either with DMSO (-), PKF 2 µM (+) or PKF 5 µM (++) for 4 hours. GAL4 reporter gene (above) or SPI1 reporter gene (middle and below). Mean with standard deviation is shown. Dots represent different samples, n = 3 independent experiments per condition. One-way ANOVA with post hoc Dunnett T3 multiple comparisons test. **e** iCisTarget motif analysis in SJTALL030263 (left) or SJTALL031201 (right) PDX samples after 24h *ex vivo* treatment with PKF 118-310 versus DMSO. Motifs in both down- (above) and upregulated genes (below) were ranked according to their respective normalized enrichment scores. SPI1 motifs are indicated in orange. P values are indicated and were calculated with a hypergeometric distribution. All results can be found in **Supplementary Data 19-22**. **f** MFI values for cKIT-PE after 24h *ex vivo* treatment of a X09 sample with PKF 118-310. P value is indicated. Two-tailed unpaired t test with Welch's correction. **g** Ex vivo treatment of spleen cells of the X09 PDX mouse and 3 control PDX mice (XB37, XB41 and XB47) (above) and J118 PDX mouse (below) with increasing doses of PKF 118-310. The J118 PDX was a *TCF7-SPI1* positive T-ALL with lineage switch to AML. The IC 50s (nM) with their respective 95% confidence intervals are: X09 494 (456 – 533), XB37 516 (462 – 565), XB41 740 (694 – 794), XB47 1137 (896 – 1838) and J118 604 (547 – 660).

Supplementary Fig. 8

a

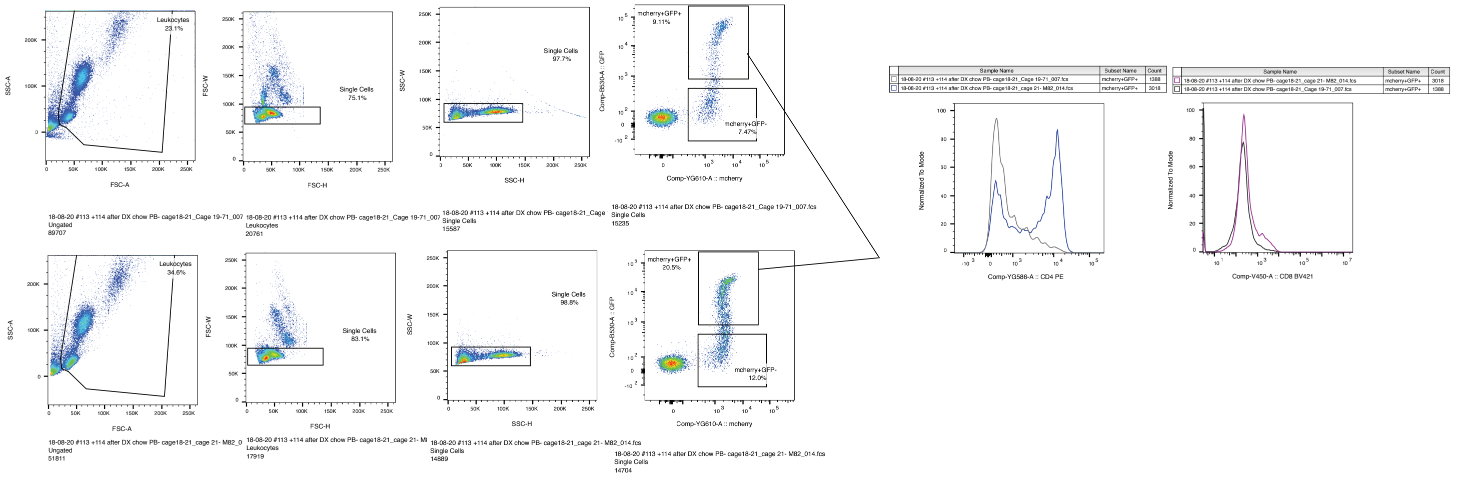

b

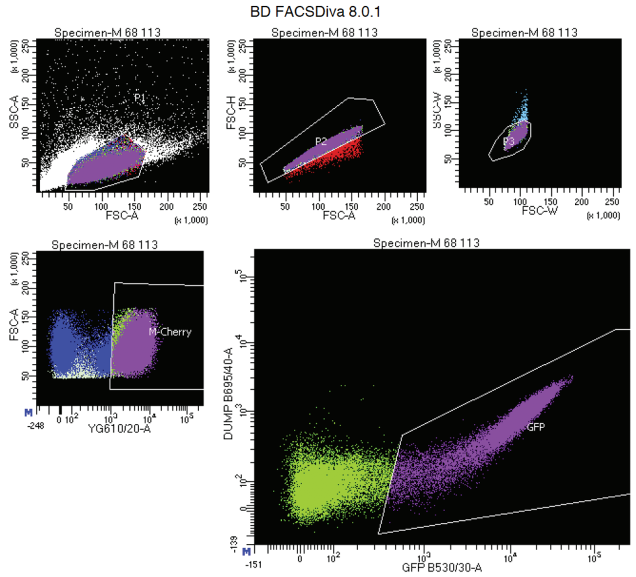

Tube: M 68 113

Population	#Events	%Parent	%Total
All Events	100,000	####	100.0
P1	67,373	67.4	67.4
P2	65,852	97.7	65.9
P3	65,644	99.7	65.6
Live	61,000	92.9	61.0
M-Cherry	47,411	77.7	47.4
GFP	29,941	63.2	29.9

Supplementary Fig. 8: Examples of gating strategies. a Representative gating strategy used for cell surface staining on peripheral cells of X09 PDX GFP/mCHERRY positive cells. **b** Representative gating strategy used to sort mCHERRY+GFP+ cells extracted from spleen at end stage for RNA extraction.

Supplementary references

1. Liu, Y. *et al.* The genomic landscape of pediatric and young adult T-lineage acute lymphoblastic leukemia. *Nat. Genet.* 49, 1211–1218 (2017).
2. Park, J. E. *et al.* A cell atlas of human thymic development defines T cell repertoire formation. *Science (80-.).* (2020)
3. Zhang, J. *et al.* The genetic basis of early T-cell precursor acute lymphoblastic leukaemia. *Nature* (2012)
