# Supplementary material for: Oncogenic cooperation between TCF7-SPI1 and NRAS(G12D) requires β-catenin activity to drive T-cell acute lymphoblastic leukemia
Source: Nat Commun. 2021 Jul 6;12:4164. doi: 10.1038/s41467-021-24442-9 (PMC8260768; doi:10.1038/s41467-021-24442-9)
Supplement: Supplementary file 2 — Description of Additional Supplementary Files [file 41467_2021_24442_MOESM2_ESM.pdf]

Description of Additional Supplementary Files

Supplementary Data 1-22:

Supplementary Data 1 - Immunophenotype patient X09

Supplementary Data 2 - Immunophenotype patient SJTALL03263_D1

Supplementary Data 3 - Immunophenotype patient SJTALL031201_D1

Supplementary Data 4 - T-ALL patient metadata

Supplementary Data 5 - Nanopore Read Data

Supplementary Data 6 - Liu_ETP&NonETP GSEA lists

Supplementary Data 7 - ETP&TALL GSEA lists

Supplementary Data 8 - Global GSEA analysis list for single-cell data

Supplementary Data 9 - RNA-seq mutation calling variant_matrix on 123 T-ALL patients

Supplementary Data 10 - List of Antibodies used in flow cytometry assays

Supplementary Data 11 - shRNAmir Oligo sequences cloned into LT3GECIR vector

Supplementary Data 12 - qPCR primers

Supplementary Data 13 - RNAseq_Zhang_signature

Supplementary Data 14 - Targeted PCR primers used to amplify TCF7-SPI1 fusion from 10x cDNA library

Supplementary Data 15 - iCisTarg IndMouse-UP

Supplementary Data 16 - iCisTarg IndMouse-DWN

Supplementary Data 17 - iCisTarg X09 PKF-UP

Supplementary Data 18 - iCisTarg X09 PKF-DWN

Supplementary Data 19 - iCisTarget results for significantly UP-regulated Genes-JTALL03263_D1 (TB-16-05839) (M100) treated with 2 uM PKF 24h

Supplementary Data 20 - iCisTarget results for significantly DOWN-regulated Genes-SJTALL03263_D1 (TB-16-05839) (M100) treated with 2 uM PKF 24h

Supplementary Data 21 - iCisTarget results for significantly UP-regulated Genes-

SJTALL031201_D1 (TB-19-01906) (M101) treated with 2 uM PKF 24h

Supplementary Data 22 - iCisTarget results for significantly DOWN-regulated Genes-

SJTALL031201_D1 (TB-19-01906) (M101) treated with 2 uM PKF 24h
