# Supplementary material for: Oncogenic cooperation between TCF7-SPI1 and NRAS(G12D) requires β-catenin activity to drive T-cell acute lymphoblastic leukemia
Source: Nat Commun. 2021 Jul 6;12:4164. doi: 10.1038/s41467-021-24442-9 (PMC8260768; doi:10.1038/s41467-021-24442-9)
Supplement: Supplementary file 4 — Reporting summary [file 41467_2021_24442_MOESM4_ESM.pdf]

nature research

Corresponding author(s): Charles de Bock, Jan Cools

Last updated by author(s): 20/05/2021

Reporting Summary

Nature Research wishes to improve the reproducibility of the work that we publish. This form provides structure for consistency and transparency in reporting. For further information on Nature Research policies, see Authors & Referees and the Editorial Policy Checklist .

Statistics

For all statistical analyses, confirm that the following items are present in the figure legend, table legend, main text, or Methods section.

n/a	Confirmed	
☐	☒	The exact sample size (n) for each experimental group/condition, given as a discrete number and unit of measurement
☐	☒	A statement on whether measurements were taken from distinct samples or whether the same sample was measured repeatedly
☐	☒	The statistical test(s) used AND whether they are one- or two-sided<br>*Only common tests should be described solely by name; describe more complex techniques in the Methods section.*
☐	☒	A description of all covariates tested
☐	☒	A description of any assumptions or corrections, such as tests of normality and adjustment for multiple comparisons
☐	☒	A full description of the statistical parameters including central tendency (e.g. means) or other basic estimates (e.g. regression coefficient) AND variation (e.g. standard deviation) or associated estimates of uncertainty (e.g. confidence intervals)
☐	☒	For null hypothesis testing, the test statistic (e.g. F, t, r) with confidence intervals, effect sizes, degrees of freedom and P value noted<br>*Give P values as exact values whenever suitable.*
☒	☐	For Bayesian analysis, information on the choice of priors and Markov chain Monte Carlo settings
☒	☐	For hierarchical and complex designs, identification of the appropriate level for tests and full reporting of outcomes
☒	☐	Estimates of effect sizes (e.g. Cohen's d, Pearson's r), indicating how they were calculated

Our web collection on statistics for biologists contains articles on many of the points above.

Software and code

Policy information about availability of computer code

Data collection	Not applicable
Data analysis	All pipelines and software packages (including versions) used for analysis of data are listed and detailed within the methods section. This includes the following:<br>FastQC (v0.11.9)<br>Bowtie2 (v2.3.5.1)<br>Tophat2 (v2.1.1)<br>VarScan (v2.4.4)<br>BreakDancer (v1.1_20100719)<br>FusionCatcher (v1.33), IGV (v2.8.2)<br>Minimap2 (v2.17-r941)<br>FlowJo (v10.6.1)<br>qbase+ software (v3.2)<br>TrimGalore (v0.6.6)<br>STAR (v.2.7.5c),<br>HTSeq (v0.12.4)<br>GENCODE (v22)<br>arriba (v1.2.0),<br>DeSeq2 R package (v1.22.0)<br>fgsea R package (v1.14.0)<br>molecular signatures database (v7.2)<br>ensemble variant effect predictor (VEP) (Ensembl release 103)<br>HISAT2 (v2.1.0)

iCisTarget (v2015),
CellRanger v3.0.2,
Seuratv3 R package (v3.2.3)
DropletUtils package (v1.4.3)
Harmony package (v1.0)
clustree R package(v0.4.1)
ComplexHeatmap Package v2.0.0
MSigdb v7.4, Guppy (v4.0.11)
Canu (version 1.8; parameters: -Overlapper=minimap -minReadLength=500 -minOverlapLength=100 -genomeSize=1k -nanopore-raw -stopOnReadQuality=false)
Minimap2 (version 2.17-r943-dirty)
Racon (version 1.3.3; parameters: -u -w 200 -m 8 -x -1 -g -4)
Gencode v34
Graphpad v9.0.2 (134)

Custom R Script: (zenodo repository: 10.5281/zenodo.4756105)

For manuscripts utilizing custom algorithms or software that are central to the research but not yet described in published literature, software must be made available to editors/reviewers. We strongly encourage code deposition in a community repository (e.g. GitHub). See the Nature Research guidelines for submitting code & software for further information.

Data

Policy information about availability of data

All manuscripts must include a data availability statement. This statement should provide the following information, where applicable:
- Accession codes, unique identifiers, or web links for publicly available datasets
- A list of figures that have associated raw data
- A description of any restrictions on data availability

The data have been deposited within the European Genome-phenome Archive (EGA), which is hosted by the EBI and the CRG. Accession number EGAS00001005097. The Seki et al RNA-seq data are available at the National Bioscience Database Center, Japan Science and Technology Agency (ID: JGAD00000000090). Processed single cell data together with custom R-scripts used have been deposited with the Zenodo Biorepository (https://10.5281/zenodo.4756105). For Gene Set Enrichment Analysis, Molecular Signatures Database was used (http://www.gsea-msigdb.org/gsea/msigdb/index.jsp.). All other relevant data are available from the corresponding authors on request.

Field-specific reporting

Please select the one below that is the best fit for your research. If you are not sure, read the appropriate sections before making your selection.

☒ Life sciences ☐ Behavioural & social sciences ☐ Ecological, evolutionary & environmental sciences

For a reference copy of the document with all sections, see nature.com/documents/nr-reporting-summary-flat.pdf

Life sciences study design

All studies must disclose on these points even when the disclosure is negative.

Sample size

For Figure 4B, the primary conditional bone marrow transplant with the TCF7-SPI1 construct was 5 mice - carried out in a single cohort. For the TCF7-SPI1_P2A_NRAS G12D and NRASG12D only there are 9 mice in total and were two independent cohorts of (n=4, n=5 for TCF7-SPI1_P2A_NRAS G12D; n=5, n=4 for the NRASG12D). For the secondary transplant recipients there are 5 mice for the NRAS only, and there were 6 recipients for TCF7-SPI1_P2A_NRAS G12D, where each was a single cohort. No power analysis was carried out for these experiments. For figure 5B, these are the recipients for a single bone marrow transplant into 5 or 6 recipients, as a single cohort experiment side-by-side. No power analysis was carried out. For figure 6B, 12 mice were used per shRNAmir construct of which 8 were placed on Doxycycline chow and 4 were placed on normal chow. No power analysis was carried out. Cohort mouse numbers for each experiment were based on our previous studies used to analyse leukemogenesis after Bone Marrow Transplant or within transgenic mouse models (see de Bock et al, Cancer Discovery 2018; DOI: 10.1158/2159-8290.; Habets et al, Science Translation Medicine 2019, DOI: 10.1126/scitranslmed.aau6246; Vanden Bempt et al, Cancer Cell 2018, DOI: 10.1016/j.ccell.2018.07.007.)

Data exclusions

In Figure 4B, 2 mice were excluded for the TCF7-SPI1_P2A_NRAS G12D primary transplants (2 mice from cohort 1 and 1 mouse from cohort 2 as there was no engraftment e.g. because of missed injection) For the NRAS only, one mouse was excluded due to no engraftment. In figure 4C, it was not possible to weigh all spleens/thymus' if a recipient mouse died in the cage overnight. For example, there are only 7 thymus weights for the NRAS only mice. As noted in the methods, the mouse was considered "event" when WBC >20,000. However, for NRAS only mice, the WBC rarely rose to this level and the surrogate marker was rapid weight loss in 24-36 hours likely due to the enlarged thymus blocking the oesophagus.

Replication

The bone marrow transplants in figure 4 concerning the TCF7-SPI1_P2A_NRAS G12D and NRAS G12D are two independent replicates with independent donor/recipient mice being used (i.e. two independent cohorts). The remainder and the secondary transplants are a single independent experiment with multiple mice. For the in vitro luciferase work, all data is presented in figure 5 where data represents technical replicates with biological replicates for these experiments presented in the supplementary figure as described in the manuscript. The replication for Figure 6B luciferase data is also provided in supplementary data. Cell growth experiments in figure 3, data represents three

	wells for each construct followed over time and each well measured once for each data collection point. For the inducible mouse model studies for doxycycline chow induced knockdown of the fusion undertaken during the revision period, a total of 12 mice were used in the experiment with 8 mice receiving dox-chow in each cohort. This experiment in total is a a single biological replicate with 8 technical replicates. Figure 5B is a single bone marrow transplant where the recipients were technical replicates all receiving the same transduced hematopoietic and stem progenitor cells. For Figure 6, the inducible knockdown experiment was a single transplant where PDX cells were injected into 12 mice (technical replicates) for each shRNAmir. This experiment was not independently repeated.
Randomization	Mice were allocated retroviral constructs on a cage per cage basis. There was no formal randomization. For the bone marrow transplants presented in Figure 4 and Figure 5, no formal randomization was undertaken and not relevant for these experiments. Covariates such as sex, age and mean weights were controlled for across the cages. For Inducible mouse experiments (Figure 6), mice were allocated per group based on percentage engraftment was normalised across Dox-Chow and Normal Chow groups to ensure no difference in engraftment occurred between the two groups.
Blinding	There was no blinding carried out during the study. To complete the analysis of the in vitro cell work correctly, there was no blinding of growth curves or luciferase expression vector experiments to complete the analysis, with sample information and corresponding treatments known to investigators. For in vivo studies, documentation outlining what mice received which retroviral construct is mandated as part of the ethics and monitoring process. All scientists were therefore aware of which mice had which construct. Similarly, for the doxycycline treatment of mice, this chow is provided on a cage per cage basis preventing investigators being blinded to the treatments.

Reporting for specific materials, systems and methods

We require information from authors about some types of materials, experimental systems and methods used in many studies. Here, indicate whether each material, system or method listed is relevant to your study. If you are not sure if a list item applies to your research, read the appropriate section before selecting a response.

Materials & experimental systems

n/a	Involved in the study
☐	☒ Antibodies
☐	☒ Eukaryotic cell lines
☒	☐ Palaeontology
☐	☒ Animals and other organisms
☐	☒ Human research participants
☒	☐ Clinical data

Methods

n/a	Involved in the study
☒	☐ ChIP-seq
☐	☒ Flow cytometry
☒	☐ MRI-based neuroimaging

Antibodies

Antibodies used

We have provided an independent table as part of the supplementary data with all antibodies used in the flow cytometry analysis.

NRAS (Abcam, Ab55391), (dilution 1:1000)
TCF1 (Thermo Fisher Scientific, MA5-14965), (dilution 1:1000)
PU.1 (Abcam-76542), (dilution 1:1000)
beta-actin (Sigma-Aldrich, A1978), (dilution 1:1000).

Human Antibodies for staining patient X09:

Target	Clone	Color	Company	Cat no.	Dilutions Used	Lot#
CD1a	HI149	PE-Cy7	eBioscience	25-0019-42	1:100	Not Available
CD2	RPA-2.10	FITC	eBioscience	11-0029-41	1:100	Not Available
CD3	OKT3	PerCP-Cy5.5	eBioscience	45-0037-42	1:50	Not Available
CD4	OKT4	FITC	eBioscience	11-0048-42	1:50	Not Available
CD7	4H9	PE	eBioscience	12-0078-41	1:200	Not Available
CD8a	HIT8a	PE	eBioscience	12-0089-42	1:200	Not Available
CD34	581	PE-Cy7	BD	560710	1:50	Not Available
CD117	YB5.B8	APC	BD	550412	1:20	Not Available
HLA-DR	L243	APC-H7	BD	641411	1:20	Not Available

Human antibodies for the staining of X09 patient-derived xenografts:

Target	Clone	Color	Company	Cat no.	Dilution	Lot#
CD3	OKT3	APC	Biolegend	317318	1:25	B317481
CD3	SK7	APC	Biolegend	344812	1:50	B276322
CD4	OKT4	PE	Biolegend	317410	1:100	B264363
CD4	SK3	PE-Cy7	Biolegend	344612	1:200	B297801
CD8	HIT8a	BV421	BD	740078	1:200	0171570
c-Kit (CD117)	104D2	PE-Cy7	eBioscience	25-1178-42	1:200	2162008
CD7	M-T701	BV605	BD	740392	1:200	0171556

CD2	RPA-2.10	APC-ef780	eBioscience	47-0029-42	1:100	2150725
CD1a	HI149	BV480	BD	566147	1:100	0022127
CD45	2D1	APC	eBioscience	17-9459-42	1:50	2034153
c-Kit (CD117)	104D2	PE	eBioscience	12-1178-42	1:200	E11779-1634

Mouse antibodies:

Target	Clone	Color	Company	Cat no.	Dilution	Lot#
CD3e	145-2C11	PE	Miltenyi Biotec	130-102-600	1:50	5160914218
CD4	RM4-5	PerCP-Cy5.5	eBioscience	45-0042-80	1:500	E08292-1633
CD8	53-6.7	APC-Cy7	eBioscience	47-0081-82	1:100	1911422
c-Kit (CD117)	2B8	APC	BD	553356	1:500	26530
CD45	30-F11	FITC	eBioscience	11-0451-82	1:100	2122625

Validation

All used antibodies were commercially available conjugated antibodies which were validated by the manufacturer for the intended use in flow cytometry; validation statements are available on the website of the manufacturer on the product page of each antibody. These are summarised below from the manufacturer's websites for each antibody:.

CD1a Monoclonal Antibody (HI149), PE-Cyanine7, eBioscience™: #25-0019-42: Applications Tested: This HI149 antibody has been pre-titrated and tested by flow cytometric analysis on the human MOLT-4 cell line. (https://www.thermofisher.com/antibody/product/CD1a-Antibody-clone-HI149-Monoclonal/25-0019-42)

CD2 Monoclonal Antibody (RPA-2.10), FITC, eBioscience™: This RPA-2.10 antibody has been pre-titrated and tested by flow cytometric analysis of normal human peripheral blood cells. (https://www.thermofisher.com/antibody/product/CD2-Antibody-clone-RPA-2-10-Monoclonal/11-0029-41)

CD3 Monoclonal Antibody (OKT3), PerCP-Cyanine5.5, eBioscience™: This OKT3 antibody has been pre-titrated and tested by flow cytometric analysis of normal human peripheral blood cells. (https://www.thermofisher.com/antibody/product/CD3-Antibody-clone-OKT3-Monoclonal/45-0037-42)

CD4 Monoclonal Antibody (OKT4 (OKT-4)), FITC, eBioscience™: tested by flow cytometric analysis of normal human peripheral blood cells. (https://www.thermofisher.com/antibody/product/CD4-Antibody-clone-OKT4-OKT-4-Monoclonal/11-0048-42)

CD7 Monoclonal Antibody (4H9), PE, eBioscience™:This 4H9 antibody has been pre-titrated and tested by flow cytometric analysis of normal human peripheral blood cells. (https://www.thermofisher.com/antibody/product/CD7-Antibody-clone-4H9-Monoclonal/12-0078-42)

CD8a Monoclonal Antibody (HIT8a), PE, eBioscience™: This HIT8a antibody has been pre-titrated and tested by flow cytometric analysis of normal human peripheral blood cells. (https://www.thermofisher.com/antibody/product/CD8a-Antibody-clone-HIT8a-Monoclonal/12-0089-42)

PE-Cy™7 Mouse Anti-Human CD34 (BD biosciences): Reactivity Human (QC Testing), Application Flow cytometry (Routinely Tested) (https://www.bdbiosciences.com/us/applications/research/stem-cell-research/cancer-research/human/pe-cy7-mouse-anti-human-cd34-581/p/560710)

Anti-HLA-DR APC-H7 Clone L243 (CE/IVD) (BD biosciences) Reactivity Human (QC Testing) Application Flow cytometry (Routinely Tested) . Immunogen: Human lymphoblastoid B-cell line RPMI 8866.9 (https://www.bdbiosciences.com/eu/reagents/clinical/reagents/single-antibodies/anti-hla-dr-apc-h7-l243/p/641411)

CD3e 145-2C11 PE Miltenyi Biotec 130-102-600 - Validated through staining of mouse splenocytes and epitope specificity of compared with other known clones recognizing the same antigen in a competition assay. Cells were incubated with an excess of purified unconjugated CD3ε (145-2C11) antibody followed by staining with fluorochrome-conjugated antibodies of other known clones against the same marker. (https://www.miltenyibiotec.com/US-en/products/cd3e-antibody-anti-mouse-145-2c11.html#pe:30-ug-in-1-ml)

CD4 Monoclonal Antibody (RM4-5), PerCP-Cyanine5.5, eBioscience™: This RM4-5 antibody has been tested by flow cytometric analysis of mouse thymocytes and splenocytes. (https://www.thermofisher.com/antibody/product/CD4-Antibody-clone-RM4-5-Monoclonal/45-0042-82)

CD8a Monoclonal Antibody (53-6.7), APC-eFluor 780, eBioscience™: This 53-6.7 antibody has been tested by flow cytometric analysis of mouse splenocytes (https://www.thermofisher.com/antibody/product/CD8a-Antibody-clone-53-6-7-Monoclonal/47-0081-82)

APC Rat Anti-Mouse CD117 Clone 2B8 (RUO) #553356 BD Bioscience: Reactivity Mouse (QC Testing) Application Flow cytometry (Routinely Tested). Immunogen:Mouse Bone Marrow Mast Cells (https://www.bdbiosciences.com/us/applications/research/stem-cell-research/cancer-research/mouse/apc-rat-anti-mouse-cd117-2b8/p/553356)

PE Mouse anti-Human CD4, Clone OKT4, Biolegend #317410. This antibody has been tested by flow cytometric analysis of mouse thymocytes. (https://www.biolegend.com/en-us/search-results/pe-anti-human-cd4-antibody-3654?GroupID=GROUP28)

BV-421 Mouse anti-Human CD8a, Clone HIT8a, BD 740078. This antibody has been tested by flow cytometric analysis of mouse thymocytes. (https://www.bdbiosciences.com/us/reagents/research/antibodies-buffers/immunology-reagents/anti-human-antibodies/cell-surface-antigens/bv421-mouse-anti-human-cd8-hit8a/p/740078)

PE-cyanine7 Mouse anti-human CD117, Clone 104D2, Lot#2162008. This antibody has been pre-titrated and tested on human peripheral blood cells. (https://www.bdbiosciences.com/us/applications/research/stem-cell-research/cancer-research/human/pe-cytrade7-mouse-anti-human-cd117-104d2/p/339195)

Eukaryotic cell lines

Policy information about cell lines

Cell line source(s)	Mel888 cells were a gift from Lionel Larue, Normal and Pathological development of melanocytes lab, Institut Curie, France. They are also known as 888-Mel and were originally purchased from ATCC. The 293T and Ba/F3 cells were from DSMZ. The Ba/F3-Cre line is a stable derivative of Ba/F3 cells that were transduced with MSCV-Cre recombinase-Puromycin for the stable expression of Cre-recombinase. These were made in house within the laboratory of Prof Jan Cools. The Pro-T primary cell line is made in house from bone marrow stem cells as outlined in the methods section.
Authentication	The Mel888 cells were not authenticated. The 293T and Ba/F3 cells were authenticated using STR analysis.
Mycoplasma contamination	293T, Mel888 and Ba/F3 cells were all Mycoplasma negative.
Commonly misidentified lines (See ICLAC register)	No commonly misidentified cell lines were used in this study

Animals and other organisms

Policy information about studies involving animals; ARRIVE guidelines recommended for reporting animal research

Laboratory animals	C57BL/6 female mice as recipients for bone marrow transplants. Male CD2-Cre on a C57BL/6 background was used as a donor mouse for the bone marrow transplant experiments. NOD.Cg-Prkdcscid Il2rgtm1Wjl/SzJ (NSG) mice or (NOD.Cg-Rag1tm1Mom Il2rgtm1Wjl/SzJArc 9NRG) mice were used for the PDX models and were always female. In all cases the mice used as donors for bone marrow transplants were aged between 6-8 weeks at time of harvest. Recipient mice for bone marrow transplants were between 8-11 weeks. For NRG and NSG mice, at time of injection ages were between 8-11 weeks. For housing, mice were held under PC2 conditions in individually ventilated caging with a maximum of 6 or a minimum of 2 mice per cage. Dimensions for mice cages: 369 X 156 X 132 mm, with a floor area of 440 cm2. The mice were maintained in a protected and controlled environment. The animal facility was barrier protected with the air HEPA filtered and the room maintained at positive pressure and at a temperature 22 +/- 1 degree Celsius. Sterile feed and water were provided ad libitum. The light in the animal facility was on a 12h cycle. All experimental work was conducted in biosafety or cytotoxic cabinets to further protect the animals from microbiological threat.
Wild animals	No wild animals were used in the study
Field-collected samples	No field collected samples were used in the study
Ethics oversight	Animal experiments were approved by the Ethical committee on animal experimentation of KU Leuven. The animal work carried out at the Children's Cancer Institute was approved by the Animal Care and Ethics Committee, University of New South Wales (Approval 20/6B)

Note that full information on the approval of the study protocol must also be provided in the manuscript.

Human research participants

Policy information about studies involving human research participants

Population characteristics	Patient X09, age 6, Male (KU Leuven). - sample was collected within Pediatric Hemato-oncology department of the University Hospital, Leuven, Belgium at the time of diagnosis on protocol S57176 by the ethical committee University of Leuven. Written informed consent was obtained in accordance with the Declaration of Helsinki. For patients SJTALL030263 and SJTALL031201 - sex and age de-identified (see meta data in supplementary table S4) - and were patients part of ongoing clinical trials at St. Jude and other participating institution and informed consent had been obtained. Use of samples was approved by UNSW Human Ethics #HC200562 and St Jude Tissue Repository #20011. Data associated with patients in the Seki et al paper was obtained from the National Bioscience Database Center Data, Japan and approved under UNSW Human Ethics #HC200558
Recruitment	This study did not recruit any patients. All experiments were performed on samples collected from University Hospital, Leuven or from samples deposited in the St Jude Biorepository. Written and informed consent and IRB/UNSW ethics approval occurred prior to use.
Ethics oversight	Approved by KU Leuven: Ethics #S57176, UNSW Human Ethics #HC200562 and #HC200558

Note that full information on the approval of the study protocol must also be provided in the manuscript.

Flow Cytometry

Plots

Confirm that:

- [x] The axis labels state the marker and fluorochrome used (e.g. CD4-FITC).
- [x] The axis scales are clearly visible. Include numbers along axes only for bottom left plot of group (a 'group' is an analysis of identical markers).
- [x] All plots are contour plots with outliers or pseudocolor plots.
- [x] A numerical value for number of cells or percentage (with statistics) is provided.

Methodology

Sample preparation	For peripheral blood from mice for WBC analysis, blood was collected into microvette tubes containing EDTA and then underwent red cell lysis to remove excess erythrocytes. WBC are then washed in PBS (2X) and then stained with antibodies according to manufacturers instructions. For blast cells within the thymus and/or spleen, organs were crushed through a 40 micron filter, treated with red cell lysis buffer to remove erythrocytes. Then washed 2X with excess PBS. Samples were refiltered through a 40 micron filter to remove any residual clumps/dead cells. These were then stained with the antibodies according to manufacturers recommended concentrations in the dark at 4 degrees (time ranged from 30 min to 60 min). All samples were washed 1X with PBS prior to analysis on instruments.
Instrument	MACSQuant VYB (Miltenyi Biotec) or FACS Verse, FACSAriaII and Fortessa (BD).
Software	FlowJo (Treestar) was used for all analysis
Cell population abundance	For Figure 6e, f and supplementary figure 6b used RNA that was sorted from mCHERRY+GFP+ cells. The abundance of this population was between 9-20% in different mice. Gating strategy is for sorting is equivalent to fig. 6c. Example gating is provided in Supplementary Figure S8
Gating strategy	Figure 1: Flow Cytometry was undertaken in the diagnostic laboratory on the primary patient sample at diagnosis and presented. Single cells were gated using FSC-A and SSC-A to gate cells. Doublet discrimination was undertaken by using SSC-W vs SSC-H and FSC-W vs FSC-H. Gates are set by their inhouse diagnostic framework to indicate positivity/negativity for each antibody used.<br><br>Figure 4E: Gating using FSC-A /SSC-A was carried out to include all cells except debris that were removed through threshold setting. Doublet discrimination was undertaken by using SSC-W vs SSC-H and FSC-W vs FSC-H. These were then gated based on fluorescence of GFP-A vs. BFP-A and then gating is outlined in the figure. The positivity of cells with fluorescence cell markers was based on matched isotype controls.<br><br>Figure 5D: Gating using FSC-A /SSC-A was carried out to include all cells except debris that were removed through threshold setting. Doublet discrimination was undertaken by using SSC-W vs SSC-H and FSC-W vs FSC-H. GFP positive cells are determine against non-GFP cells. This is the gating strategy used on all the GFP positive cells and then subsequently for CD4 and CD8 markers with positivity of marker expression based on matched isotype controls.<br><br>Figure 6C: Gating using FSC-A /SSC-A was carried out to include all cells except debris that were removed through threshold setting. Doublet discrimination was undertaken by using SSC-W vs SSC-H and FSC-W vs FSC-H. Positivity of GFP or mCHERRY cells based on non-transduced cells. CD4, CD8, cKIT and CD2 expression was based on isotype controls.<br><br>Supplementary Figure S8 provides example of gating used for staining for Figure 6C showing FSC-A/SSC-A gates, singlet cell gating and then subsequent GFP/mCHERRY and CD4/CD8 histograms. Figure S8B illustrates gating used to sort and expand X09 PDX cells transduced with the inducible lentiviral construct.

- [x] Tick this box to confirm that a figure exemplifying the gating strategy is provided in the Supplementary Information.
